# Acceptance and Commitment Therapy (ACT) for people with advanced progressive illness, their caregivers and staff involved in their care: A scoping review

**DOI:** 10.1177/02692163231183101

**Published:** 2023-07-25

**Authors:** Tilly Gibson Watt, David Gillanders, Juliet A Spiller, Anne M Finucane

**Affiliations:** 1 University of Edinburgh Medical School, University of Edinburgh, Scotland, UK; 2 Clinical Psychology, School of Health in Social Science, University of Edinburgh, Scotland, UK; 3 Marie Curie Hospice Edinburgh, Edinburgh, Scotland, UK

**Keywords:** Acceptance and Commitment Therapy, palliative care, caregivers, bereavement, staff, terminally ill, scoping review

## Abstract

**Background:**

People with an advanced progressive illness and their caregivers frequently experience anxiety, uncertainty and anticipatory grief. Traditional approaches to address psychological concerns aim to modify dysfunctional thinking; however, this is limited in palliative care, as often concerns area valid and thought modification is unrealistic. Acceptance and Commitment Therapy is a mindfulness-based behavioural therapy aimed at promoting acceptance and valued living even in difficult circumstances. Evidence on its value in palliative care is emerging.

**Aims:**

To scope the evidence regarding Acceptance and Commitment Therapy for people with advanced progressive illness, their caregivers and staff involved in their care.

**Design:**

Systematic scoping review using four databases (Medline, PsychInfo, CINAHL and AMED), with relevant MeSH terms and keywords from January 1999 to May 2023.

**Results:**

1,373 papers were identified and 26 were eligible for inclusion. These involved people with advanced progressive illness (*n* = 14), informal caregivers (*n* = 4), palliative care staff (*n* = 3), bereaved carers (*n* = 3), and mixed groups (*n* = 2). Intervention studies (*n* = 15) showed that Acceptance and Commitment Therapy is acceptable and may have positive effects on anxiety, depression, distress, and sleep in palliative care populations. Observational studies (*n* = 11) revealed positive relationships between acceptance and adjustment to loss and physical function.

**Conclusion:**

Acceptance and Commitment Therapy is acceptable and feasible in palliative care, and may improve anxiety, depression, and distress. Full scale mixed-method evaluation studies are now needed to demonstrate effectiveness and cost-effectiveness amongst patients; while further intervention development and feasibility studies are warranted to explore its value for bereaved carers and staff.

**What is already known about the topic?**

- There is a rapidly growing evidence base for the use of Acceptance and Commitment Therapy in the management of psychiatric disorders, chronic conditions and stress.
- Evidence on the use of Acceptance and Commitment Therapy for people with advanced cancer, concluded that it may be beneficial in improving psychological symptoms.

**What this paper adds?**

- Acceptance and Commitment Therapy interventions are feasible and acceptable for people with advanced progressive illness; and may improve anxiety, depression, distress, sleep, and quality of life.
- Evidence on Acceptance and Commitment Therapy for people with advanced progressive diseases other than cancer is lacking; and there are very few studies on its use or value for bereavement support.

**Implications for practice, theory or policy**

- Acceptance and Commitment Therapy has the potential to help manage psychological concerns in palliative care settings, though further evidence of effectiveness is warranted.
- Training for palliative care staff in the principles of Acceptance and Commitment Therapy could increase access to evidence-based psychological support for people with advanced progressive illness.

## Introduction

Palliative care is an approach that aims to improve the quality of life of people who are facing problems associated with advanced progressive illness, and their families.^1^ Existential distress is frequently a concern for people with an advanced progressive illness, irrespective of culture or type of disease.^1^ Anxiety, worry, uncertainty, loss of control and social role are common.^2–5^ Some people find it hard to engage in meaningful activities and relationships, may lose hope, and experience anticipatory grief .^6,7^ Regrets can occur where there is discrepancy between how people want things to happen and how they actually happen. These include regrets due to unfinished role responsibilities and broken relationships.^4,6^

Caregivers of people with an advanced progressive illness can also experience depression, anticipatory grief, loneliness, and a disturbance in their sense of meaning and purpose.^8^ Increases in psychological symptoms such as anxiety and depression, as well as physical symptoms such as fatigue and pain due to their caregiver role is also common.^9^ Up to 40% of caregivers are at moderate or high risk of prolonged grief disorder following bereavement.^10^ Bereavement support needs increased during the COVID-19 pandemic, with many people reporting severe grief and difficulties accessing support.^11,12^ Evidence based interventions that help patients and caregivers recognise and manage psychological distress in the context of deteriorating health and bereavement are vital for high quality palliative care.^4,13^

Staff caring for people with an advanced progressive illness are at risk of stress, distress and burnout. This is partly due to the typical demands associated with working in healthcare such as unmanageable workloads and staff shortages, but also because of regular exposure to death, grief and loss.^14–19^ Over one-third of palliative care physicians met the criteria for burnout during the COVID-19 pandemic, further highlighting the need for interventions to support mental wellbeing amongst staff.^20^

Acceptance and Commitment Therapy (ACT) is a form of Cognitive Behavioural Therapy that has its roots in radical behaviourism and emphasises how we respond to thoughts and feelings, rather than trying to alter the meaning of the situation as in traditional cognitive therapy.^21^ It supports people to identify what is important to them, and to take action to enable them to live a life of meaning and value.^22^ Instead of focussing on emotion regulation and symptom reduction, Acceptance and Commitment Therapy promotes acceptance of difficult thoughts and feelings, and acting in ways that are aligned with meaningful and valued living, even in the presence of difficult thoughts, feelings and circumstances.^23^ This different focus gives Acceptance and Commitment Therapy a clear rationale and potential to improve wellbeing and quality of life of those impacted by advanced progressive illness, including caregivers and staff.

The Acceptance and Commitment Therapy model consists of six interdependent and overlapping processes. These processes are: (1) Acceptance – making space for challenging thoughts and emotions versus suppression or avoidance; (2) Defusion – stepping back from unhelpful thoughts and emotions to reduce their influence; (3) Contact with the present moment – maintaining flexible awareness of the present versus being overly influenced by concerns about the past or future); (4) Self as context – flexible perspective taking on our own self narratives versus being dominated by a rigidly held set of beliefs about who we are; (5) Values – identification of personal qualities and behaviours for a meaningful life versus dominance by expectations; (6) Committed Action – effective actions guided by values versus inaction or impulsivity.^24^

There is a rapidly growing evidence base for the efficacy of Acceptance and Commitment Therapy in the treatment of a broad range of disorders and conditions, with recent systematic reviews describing its potential value for people with mood disorders, anxiety disorders, chronic illness, cancer survivorship, audiological problems, eating disorders, neurodevelopmental disorders, autism, sleep disorders as well as student wellbeing.^25–36^ Acceptance and Commitment Therapy can also be effective in improving general and work-related distress amongst health care professionals,^37^ and for improving depression and quality of life amongst caregivers.^28^ However, despite the emerging evidence base for the effectiveness of Acceptance and Commitment Therapy across a range of populations and contexts, we identified only three reviews focused specifically on advanced progressive illness or bereavement.^38–40^ Two of these reviews concluded that Acceptance and Commitment Therapy may be beneficial in improving quality of life and alleviating symptoms of anxiety and distress amongst patients with advanced cancer^38,39^ However, both reviews excluded studies of people with advanced illnesses other than cancer, and considered evidence based on RCTs or quasi-experimental studies only.^38^ Neither review sought to explore the potential value of Acceptance and Commitment Therapy for caregivers or staff involved in the care of people with advanced progressive illness. Consequently, given the emerging use of Acceptance and Commitment Therapy in palliative care^41^ and its potential to improve outcomes for patients, caregivers and staff, we sought to determine the scope of the literature on Acceptance and Commitment Therapy for a broadly defined palliative care population. We were keen to report on all types of evidence that might address and inform clinical practice, explore how research has been conducted, identify characteristics related to delivering Acceptance and Commitment Therapy in this field, examine the outcomes that have been evaluated, impacts reported and identify evidence gaps.

### Aim and research questions

Our aim was to map the evidence on Acceptance and Commitment Therapy for people with an advanced progressive illness, their caregivers (includes family members), and staff involved in their care. Specific research questions were:

1. What is the evidence for the feasibility, acceptability, and effectiveness of Acceptance and Commitment Therapy for people with a terminal or advanced progressive illness, their informal caregivers, including bereaved caregivers, and staff involved with their care?
2. How has Acceptance and Commitment Therapy has been delivered in palliative care settings (format, duration, mode of delivery, content, facilitation, barriers)?
3. What types of research designs have been undertaken and what outcomes have been measured?

## Method

### Design

A systematic scoping review was undertaken, guided by the Joanna Briggs Institute framework for scoping reviews.^42^ This type of review allows diverse types of evidence to be mapped comprehensively to identify all evidence in an emerging field.^42^ Scoping reviews are useful for examining broad areas to identify evidence gaps, clarify key concepts and report on evidence to address and inform progress in a topic area. The PRISMA-ScR guidelines were used to inform the reporting of the review.^43^ The protocol was registered on the Open Science Framework registries: (https://osf.io/tpqax).

### Eligibility criteria

We included studies regarding the use of Acceptance and Commitment Therapy for people with an advanced progressive illness, their caregivers or staff involved in their care (Table 1).

**Table 1.** Population – Concept – Context framework.

Element	Description
**Population**	People with an **advanced progressive illness** or serious life-threatening medical condition which is irreversible, and which will continue indefinitely, where there is no reasonable hope of recovery (including patients receiving hospice or palliative care) OR Informal caregivers of people with an advanced progressive illness such as family members, partners, chosen-family, close friends, or friends who are involved in providing any form of care OR Formal caregivers of people with an advanced progressive illness such as any healthcare professional involved in their care, For example doctors, nurses and healthcare assistants, allied health professionals, and clinical support staff OR Bereaved caregivers including both bereaved informal caregivers and formal caregivers (i.e. professionals) who may have experienced grief, bereavement or loss as part of their role.
**Concept**	**Acceptance and Commitment Therapy** delivered by any health care professional, including, but not limited to, physicians, registered nurses, physiotherapists, psychologists, social workers, and occupational therapist OR any study were Acceptance and Commitment Therapy processes are examined.
**Context**	**Any setting** where Acceptance and Commitment Therapy has been delivered or evaluated including hospital, hospices, care homes or community settings. This includes Acceptance and Commitment Therapy delivered online, and in individual or group contexts.

### Types of sources

We considered empirical research studies published after 1999 (when Acceptance and Commitment Therapy was first described in detail).^23^ This includes quantitative, qualitative, and mixed method studies; studies carried out in the English language. We excluded reviews, editorials, commentaries, case studies and dissertations below doctoral level; any studies which did not specify clearly whether a majority (>50%) of the population had an advanced progressive illness; any studies where the Acceptance and Commitment Therapy based intervention focused only on one component of Acceptance and Commitment Therapy (e.g. mindfulness); and any studies where Acceptance and Commitment Therapy was not a major component (<50%).

## Search strategy

An electronic search was carried out in Ovid MEDLINE, PsychInfo, AMED and CINAHL covering the period of January 1999 to 12^th^ May 2023.The search was undertaken using a combination of Medical Subject Headings (MeSH) and free text search terms for palliative care, Acceptance and Commitment Therapy and a range of terms for advanced progressive illnesses (See Supplemental File for Ovid MEDLINE search strategy). The terms used to retrieve the relevant papers were based on prior reviews and guidance from the research team’s expertise in psychological therapies and palliative care. The terms were adapted following pilot searches and varied slightly depending on the requirements of the database. ProQuest, ISRCTN and ICTRP registries were searched to identify further relevant papers. The reference lists of included papers and the reviews identified were hand-searched. Finally, all citations were exported to Endnote and duplicates were removed.

## Data screening

Titles and abstracts were screened by one reviewer (TGW) and a sub-sample (25%) were independently reviewed by a second reviewer (AF) to check consistency as the searching progressed. Full texts were reviewed by one co-author (TGW) and a sub-sample (50%) were independently reviewed by a second (AF). Any queries were discussed by the full team to determine a definite list of studies.

## Data extraction

Data were extracted by one reviewer (TGW) using a data extraction protocol developed for the purpose of the study.^44^ A second reviewer (AF) reviewed a sub-sample (five papers) to help to ensure the accuracy and congruency in the data extraction process. The extracted data was tabulated on an Excel spreadsheet according to the following: author(s), title, author(s) origin, year of publication, journal, study type, population, setting, methodology, intervention format, healthcare professional level and outcomes. Any uncertainty with data categorisation was reviewed by all co-authors. Due to the wide range of study methodologies and heterogeneous nature of data, and in keeping with the aims of a scoping review, quality appraisal was not carried out.^42^

## Data analysis and synthesis

Based on our interest in scoping evidence on Acceptance and Commitment Therapy relevant to patients, caregivers and staff, we decided on a broadly deductive approach in order to map evidence for these groups. However, during the review process, it became clear that evidence could be usefully categorised as pertaining to interventions using Acceptance and Commitment Therapy; or observation studies describing relationships between Acceptance and Commitment Therapy components and outcomes commonly assessed in palliative care. Consequently, we decided to separately report intervention and observational studies. Consequently. For intervention studies we reported data on key characteristics such as setting, mode of intervention delivery, intervention format, outcomes assessed, enrolment and retention rates as well as findings. For observational studies we reported data on study aim, methods, outcomes and key findings. To support integration, quantitative data were tabulated and analysed descriptively in MS Excel. All studies were imported into NVivo so any qualitative data could be analysed and synthesised descriptively guided by the review aims (e.g. evidence on intervention acceptability).

## Results

### Study selection

**Figure 1.**
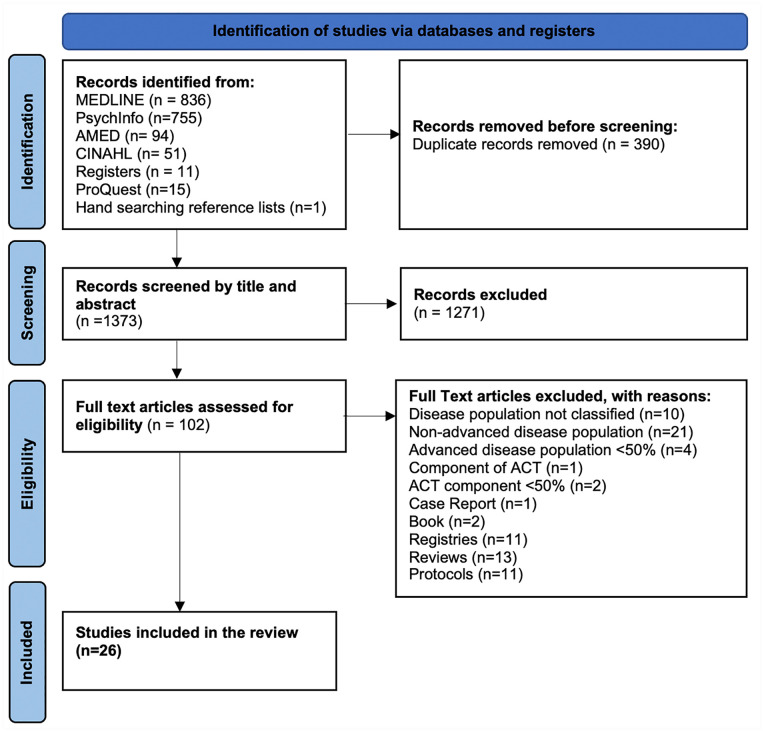
PRISMA flow chart highlighting the search strategy.

A total of 17,638 records were identified from the electronic searches of databases, registries and hand searching reference lists. Duplicates were removed using Endnote resulting in 1,373 citations, a further 1,271 irrelevant records were excluded after screening. A total of 102 articles were selected for full text review. Overall, 26 studies were included (See Figure 1).

### Overview of studies

Nearly half of the 26 studies included were conducted in the USA (*n* = 11; 42%),^45–55^ around a quarter in the UK (*n* = 6; 23%)^56–61^ and a quarter in Australia (*n* = 6; 23%).^62–67^ Articles identified were published from 2012 to 2023 (see Supplemental File: Figure 1). The publication rate accelerated during that time, with the highest number being published in 2020 (*n* = 5). Of the 26 papers included, 15 were interventions studies^16,46,48,49,52–55,57,60–63,66,68^ while 11 were observational studies (eight quantitative, two qualitative and one mixed) (see Supplemental File: Figure 3).^45,47,50,51,56,58,59,64,65,67,69^ Intervention papers included randomised controlled trials (*n* = 3; 20%),^54,63,66^ pilot randomised controlled trials (*n* = 5; 33%),^48,49,53,55,60^ single-arm intervention pilot trials (*n* = 6; 40%)^16,46,52,57,61,62^ and qualitative intervention studies (*n* = 1; 6%).^68^

### Study populations

The majority of articles examined Acceptance and Commitment Therapy for patients (*n* = 15; 58%).^16,47–50,52–54,57,58,60,65,67,69^ Of these, 10 papers were focused on people with advanced cancer.^16,47,49,50,52–54,57,58,60^ Four were focused on Acceptance and Commitment Therapy for families or caregivers^62,63,66,68^, two a mix of both patients and caregivers.^48,55^ Three were focused on staff^45,56,61^ and a further three involved people who had been bereaved.^46,51,64^ One study involved a mix of both staff and bereaved caregivers.^59^ See Supplemental File: Figure 4.

### Intervention studies

Fifteen papers evaluated an intervention.^16,46,48,49,52–55,57,60–63,66,68^ Details of the interventions can be found in Tables 2 to 4. Of these, seven involved patients^16,49,52–54,57,60^; four involved caregivers ^62,63,66,68^; two involved patients and carers ^48,55^; one involved bereaved and staff^46^ and one staff.^61^

**Table 2.** Details of intervention studies (*N* = 15 papers).

	Author (YR)	Title	Aim	Study type	Sample
1	Arch et al. 2020^16^	Acceptability, feasibility, and efficacy potential of a multimodal Acceptance and Commitment Therapy intervention to address psychosocial and advance care planning needs among anxious and depressed adults with Metastatic Cancer	To explore the use of a multi-modal Acceptance and Commitment Therapy intervention (M_ACT) to address the psychosocial and advance care planning needs of anxious and depressed patients with metastatic cancer.	Single arm intervention development and Pilot Trial	Patients *N* = 35 anxious and depressed adults with stage IV cancer.
2	Burke et al. 2014^62^	Adapting Acceptance and Commitment Therapy for parents of children with life-threatening illness: Pilot study	To pilot a novel parent targetted intervention for parents with children diagnosed with life threatening illness with the aim of reducing parental distress.	Single arm intervention pilot trial	Caregivers *N* = 19 parents of children who had a life threatening illness diagnosis
3	Davis et al. 2020^63^	Feasibility randomised controlled trial of a self-help acceptance and commitment therapy intervention for grief and psychological distress in carers of palliative care patients	To assess the feasibility of an Acceptance and Commitment Therapy intervention for carers of patients receiving palliative care. (i) Test the feasibility of recruitment, attrition and data collection procedures; (ii) determine intervention engagement; (iii) evaluate acceptability of the intervention and (iv) evaluate preliminary effectiveness of the intervention on increasing acceptance and valued living while reducing grief and distress at 1-month follow-up.	RCT Intervention group (*n* = 53) Control group (*n* = 53)	Caregivers – active and bereaved informal caregivers randomised *N* = 106: 55 completed baseline; 44 completed 1 month follow-up; 29 completed 6-month follow-up.
4	Finucane et al. 2023^61^	Feasibility of RESTORE: An online Acceptance and Commitment Therapy intervention to improve palliative care staff wellbeing	To develop, and feasibility test, an online Acceptance and Commitment Therapy intervention to improve wellbeing of palliative care staff.	Single arm pilot intervention trial	Staff *N* = 25 health and social care professionals from Marie Curie Hospices in Scotland providing care to terminally ill patients and their families.
54	Hulbert-Williams et al. 2021^57^	Brief Engagement and Acceptance Coaching for Hospice Settings (the BEACHeS study): results from a Phase I study of acceptability and initial effectiveness in people with non-curative cancer	To develop and test acceptability and potential efficacy of a brief Acceptance and Commitment Therapy-based coaching intervention to support people with an incurable cancer diagnosis, at the point of referral to hospice.	Single arm intervention Pilot Trial	Patients *N* = 10 patients recently referred to hospice with non-curative cancer.
6	Johnson 2014^46^	A pilot study of Acceptance and Commitment Therapy (ACT) for Prolonged Grief Disorder (PGD)	To examine the efficacy of Acceptance and Commitment Therapy for individuals who met the criteria for Prolonged Grief Disorder.	PhD Single arm intervention pilot trial	Bereaved *N* = 2 bereaved patients with symptoms lasting longer than 6 months.
7	Kohle et al 2017^68^	User-experiences with a web-based self-help intervention for partners of cancer patients based on acceptance and commitment therapy and self-compassion: a qualitative study	To explore the user experience with a Web based self-help intervention based on Acceptance and Commitment Therapy and self-compassion among partners of cancer patients.	Qualitative	Caregivers *N* = 14 partners of cancer patients (4 curative intent, 1 patient recovered, 9 patients will probably not recover).
8	Mosher et al. 2023^55^	Acceptance and commitment therapy for patient fatigue interference and caregiver burden in advanced gastrointestinal cancer: Results of a pilot randomised trial	To examine the feasibility, acceptability, and preliminary efficacy of acceptance and commitment therapy for patient-caregiver dyads coping with advanced gastrointestinal cancer. Primary outcomes were patient fatigue interference and caregiver burden.	Pilot RCT Intervention (*n* = 20) Control (*n* = 20)	Patients and caregiver dyads *N* = 40 pairs of patients with advanced gastrointestinal cancer and their caregivers.
9	Mosher et al. 2019^48^	Acceptance and Commitment Therapy for symptom interference in advanced lung cancer and caregiver distress: a pilot randomised trial	To examine the feasibility and preliminary efficacy of telephone-based Acceptance and Commitment Therapy for symptomatic, advanced lung cancer patients and their distressed family caregivers.	Pilot RCT Intervention (*n* = 25) Control (*n* = 25)	Patients and caregiver dyads *N* = 50 pairs of patients with advanced lung cancer and their caregivers.
10	Mosher et al. 2018^49^	Acceptance and commitment therapy for symptom interference in metastatic breast cancer patients: a pilot randomised trial	To examine the feasibility and preliminary efficacy of telephone-based Acceptance and Commitment Therapy for symptom interference with functioning amongst patients with metastatic breast cancer.	Pilot RCT Intervention (*n* = 23) Control (*n* = 24)	Patients *N* = 47 patients with symptomatic metastatic breast cancer.
11	Muscara et al. 2020^66^	Effect of a Videoconference-Based Online Group Intervention for Traumatic Stress in Parents of Children with Life-threatening Illness: A Randomised Clinical Trial	To evaluate the efficacy of an Acceptance and Commitment Therapy-based group intervention, delivered using videoconferencing, to reduce post-traumatic stress symptoms in parents of children with a life-threatening illness.	RCT Intervention (*n* = 152) Control (*n* = 161)	Caregivers *N* = 313 parents of children with a recently diagnosed life threatening illness.
12	Plumb Vilardaga, et al. 2020^52^	Coping Skills Training and Acceptance and Commitment Therapy for Symptom Management: Feasibility and Acceptability of a Brief Telephone-Delivered Protocol for Patients with Advanced Cancer	To assess the feasibility and acceptability of telephone implementation of Engage (novel Coping Skills and ACT protocol) for reducing symptoms and increasing quality of life in community patients with advanced cancer.	Single Arm Intervention Pilot Trial [Feasibility]	Patients *N* = 24 patients with advanced cancer living in communities 60 mi away from academic medical centre and two rural community cancer treatment clinics.
13	Rost et al. 2012^53^	Improving psychological adjustment among late-stage ovarian cancer patients: Examining the role of avoidance in treatment	To explore the efficacy of Acceptance and Commitment Therapy and to compare its effects to that of a treatment as usual condition (consisted of CBT components).	Pilot RCT TAU^a^ (*n* = 22) ACT (*n* = 25)	Patients *N* = 47 women with Stage III or IV ovarian cancer.
14	Serfaty et al. 2019^60^	Acceptance and commitment therapy for adults with advanced cancer (CanACT): A feasibility randomised controlled trial	To explore the feasibility of recruiting people with advanced cancer into a RCT of Acceptance and Commitment Therapy vs standardised talking control and delivering Acceptance and Commitment Therapy to this population.	Pilot RCT [Feasibility] ACT (*n* = 22) Talking control (*n* = 20)	Patients *N* = 42 people with advanced cancer.
15	Wells-Di Gregorio et al. 2018^54^	Pilot randomised controlled trial of a symptom cluster intervention in advanced cancer	To evaluate a three-session acceptance based cognitive behavioural Acceptance and Commitment Therapy intervention CBT-ACT) targetting a common symptom cluster in advanced cancer (worry- insomnia- depression- fatigue).	Pilot RCT CBT-ACT (*n* = 17) Waitlist control (*n* = 11)	Patients *N* = 28 people with advanced cancer.

a TAU: Treatment as Usual.

**Table 3.** Details of interventions evaluated (*N* = 15).

	Author (YR)	Description of Intervention	Individual or group	Setting	Method of Delivery	Time points of data collection	Healthcare Professional Level^70^
1	Arch et al. 2020^16^	Alternated four in-person group sessions with three self-paced online session: • 2 h group sessions × 4 • 45-minute individual self-paced online sessions × 3 • 5-minute daily check in. Content: Core concepts of Acceptance and Commitment Therapy with focus on ACP. Adapted content based on individual’s values and goals.	Mixed	Hospital	In person and self-directed online	Pre-intervention, mid-intervention (after second session), 1-week post-intervention, 2-months post-intervention.	Level 4 Clinical Psychologist & onsite social worker.
2	Burke et al. 2014^62^	• 90-minute group sessions × 5. First four were delivered weekly, the fifth was delivered on month later. Content: Range of therapeutic activities common to Acceptance and Commitment Therapy (metaphors, self-reflection, experiential exercises) with problem solving skills training linked to individual values.	Group	Hospital	In person	Pre, post and 6-month follow up.	Not specified.
3	Davis et al. 2020^63^	Skills-based booklet and telephone support: • 1 booklet • 1 × phone call 1–2 weeks following receiving booklet. Content: Booklet and accompanying CD were based on core concepts of Acceptance and Commitment Therapy and contained psychoeducation and experiential mindfulness exercises aimed to help manage difficult thoughts and feelings, and to pursue valued based action.	Individual	Community- Home	Telephone and Booklet	Pre-intervention, mid-intervention (2 months), 6-months post-intervention.	Student Clinical Psychology PhD student.
4	Finucane et al. 2023^61^	Eight modules delivered by Microsoft Teams over an 8 weeks period. • 3× facilitated online workshops • 5× self-directed e-learning modules. Content: Core concepts of Acceptance and Commitment Therapy were assessed with the e-learning modules, content work books, homework and group workshops.	Mixed	Community-home	Videocall and Online	Pre-intervention, mid-intervention (week 4), post intervention (week 8), follow up (1 month post intervention).	Level 4 Senior Academic Psychologist.
5	Hulbert-Williams et al. 2021^57^	Five session in-person intervention. 40–60 min individual sessions (once weekly then, fifth delivered approx. 1 month after the fourth). Assessment session, followed by 3 Acceptance and Commitment Therapy session, and one final consolidation session. Content: Assessment and core concepts of Acceptance and Commitment Therapy including workability, awareness, openness, and engagement.	Individual	Hospice	In person	Pre-intervention, mid-intervention, post-intervention.	Level 4 Clinical Psychologist.
6	Johnson 2014^46^	8 sessions weekly delivered over 8 weeks (duration not specified). Content: Sessions consisted of the core principles of Acceptance and Commitment Therapy with the use of established metaphors and exercises used.	Individual	Hospital -Clinic	In person	Pre-intervention, mid-intervention, post intervention and 1 month post intervention.	Student Masters student with training in Acceptance and Commitment Therapy and working under supervision of clinical psychologist.
7	Kohle et al 2017^68^	Web based self-help and self-paced intervention consisting of 6 lessons. Content: Core Acceptance and Commitment Therapy processes and self-compassion. Each lesson contained a mindfulness exercise that could be downloaded. Option to connect with peers (other carers).	Individual	Community- Home	Online Programme	Semi-structured interviews conduct with participants on completion of the intervention.	Student Dutch Masters Student of Health Psychology.
8	Mosher et al. 2022^55^	Six 50 minute telephone sessions once weekly. Sessions 1,4,5 and 6 were delivered to both the patient and caregiver together. Sessions 2 and 3 were delivered separately. Content: All 6 core concepts of Acceptance and Commitment Therapy with an emphasis on present-moment awareness training and goal setting based on personal values.	Mixed	Community-Home	Telephone	Pre-intervention, 2-week and 3 months post intervention.	Level 2 Social Worker with experience in Acceptance and Commitment Therapy.
9	Mosher et al. 2019^48^	Six 50–60-min telephone sessions once weekly. Sessions 1,4,5 and 6 were delivered to both the patient and caregiver together. Sessions 2 and 3 were delivered separately. Content: All 6 core concepts of Acceptance and Commitment Therapy delivered through metaphors and experiential exercises (clarification of values and taking steps for valued living).	Mixed	Community- Home	Telephone	Pre-intervention, 2-week and 6-week post-intervention.	Level 2 Social Worker with experience in Acceptance and Commitment Therapy.
10	Mosher et al. 2018^49^	Telephone sessions (50–60 min) once weekly over 6 weeks. Handouts summarising content were mailed to participants alongside CDs with mindfulness exercises. Content: Covered all 6 core concepts of Acceptance and Commitment Therapy delivered through metaphors and experiential exercises (clarification of values and taking steps for valued living).	Individual	Community- Home	Telephone	Pre-intervention, 2-week and 6-week post-intervention.	Level 2 Social Worker with experience in Acceptance and Commitment Therapy.
11	Muscara et al. 2020^66^	Take a Breath (TAB) intervention consisting of a six-session intervention delivered online over 8 weeks. 90-minute group sessions (5 weekly sessions followed by one session 3 weeks post fifth session). Content: TAB covered core concepts of Acceptance and Commitment Therapy with a particular focus on mindfulness in each session. Materials (value cards. Session booklet, guided mindfulness CD) were sent to parents.	Group	Community- Home	Online (Videocall)	Parents completed questionnaires at 3 time points: • Within 4-weeks of child’s diagnosis or admission • 2-weeks before intervention commencement or 4–6 months post diagnosis • Immediately after intervention	Level 4 Two trained mental health clinicians.
12	Plumb Vilardaga, et al. 2020^52^	Intervention combined elements of Coping Skills Training and Acceptance and Commitment Therapy delivered via 45-minute telephone sessions over 4 weeks. Content: Combination of CST and Acceptance and Commitment Therapy: • Session Topics (Biopsychosocial Spiritual Model of Health, Value Guided Activity Planning, Coping with Negative Thoughts, Skills Integration) • Participants received a study workbook with audio player for mindfulness exercises.	Individual	Community- Home	Telephone and Booklet	Pre-intervention and post-intervention.	Level 4 Psychologist with more than 10 years training in Acceptance and Commitment Therapy.
13	Rost et al. 2012^53^	Twelve 60-minute in person individual sessions involving patient and therapist over 4 months period. Content: Acceptance and Commitment Therapy core concepts incorporating the key psychological processes such as mindfulness, acceptance and values.	Individual	Hospital	In person	Pre-intervention, post fourth, eighth and 12th session.	Student Clinical Psychology student.
14	Serfaty et al. 2019^60^	• Eight 60-minute individual sessions delivered weekly. • Participants were given 3-months to complete the intervention. Content: All 6 Core Acceptance and Commitment Therapy processes covered, with a focus on psychological flexibility in the latter sessions.	Individual	Hospice, therapist’s clinic, or home	In person	Pre-intervention and post-intervention.	Level 4 Therapists with 2 years of experience of using 2 years of Acceptance and Commitment Therapy. Received 2 full days of training of Acceptance and Commitment Therapy and TC in a palliative population.
15	Wells-Di Gregorio et al. 2018^54^	• 90-minute modules over 6 weeks − 2 in-person modules and one delivered by DVD. • Participants were asked to use a relaxation CD 3 × *a* day. Content: CBT components include relaxation, sleep hygiene, sleep restriction and SMART goal setting. Acceptance and Commitment Therapy components included self as context, defusion, identifying values and acceptance.	Individual	Hospital and Community- home	In person and DVD	Pre-intervention, post-intervention and 6-week after.	Level 4 Post-doctoral fellows in psychosocial oncology supervised by PI.

**Table 4.** Outcomes and findings of Acceptance and Commitment Therapy Intervention studies (*N* = 15).

	Author (YR)	Outcomes and details of these (e.g. how measured)	Enrolment rate	Retention Rate	Key findings
1	Arch et al. 2020^16^	• Anxiety and depressive symptoms assessed using Hospital Anxiety and Depression Scale. • Fear of dying assessed using Death and Dying Distress Scale. • Pain interference assessed using Rand Short Form Health Survey-36 single item pain interference question. • Sense of life meaning assessed Functional Assessment of Chronic Illness Therapy-Spirituality peace/meaning scale). • Acceptability and feasibility were assessed through enrolment, attendance, and satisfaction ratings.	69% referred to the study actually enrolled (35/51).	26/35 (74%) commenced the intervention. 24/35 (69%) completed the assessments.	• Completers showed significant reductions in anxiety, depression, and fear of dying. • Completers showed significant increases in ACP and sense of life meaning. • No significant impact on pain interference. • M-ACT is feasible, acceptable and shows potential for efficacy – a randomised trial is warranted.
2	Burke et al. 2014^62^	• Psychological flexibility assessed using three subscale scores (Cognitive Defusion, Committed Action and Acceptance) of the 30 item Parental Psychological Flexibility Questionnaire. • Mindfulness was assessed using 15 item Mindful Attention Awareness Scale. • Parent Distress was measured by the PTSD Checklist-Civilian Version.	‘23 families expressed an interest in participation’. But enrolment rate was not reported.	83% (19/23) of parents who expressed an interest started the intervention. 73% (8/11) of families involved completed the intervention.	• Completers showed significant improvements in distress, psychological flexibility, and mindfulness with medium to large effect sizes at post and follow up assessments. • Provided early indications that Acceptance and Commitment Therapy combined with problem-solving skills training is an appropriate therapeutic approach.
3	Davis et al. 2020^63^	• Feasibility and Acceptability assessed using response and attrition rates and questionnaire assessing experience. • Experiential avoidance assessed using the AAQ-II. • Valued Living assessed using the Valued Living Questionnaire. • Grief assessed using the PG-13 scale. • Anticipatory Grief assessed using PG-12 scale. • Psychological Distress assessed using the Hospital Anxiety and Depression Scale.	106/186 (57%) provided verbal consent and were randomised.	41.5% at 1-month follow up. 27% at 6-month follow-up.	• The intervention was generally feasible and viewed as acceptable to carers. • Preliminary effectiveness analyses showed at least a small effect size suggesting improvement in acceptance, valued-living, grief and psychological distress.
4	Finucane et al. 2023^61^	• Feasibility and acceptability data was assessed using response and attrition rate as well as qualitative data in the intervention follow up. • Psychological Flexibility was assessed using CompACT scale. • Self-reported perceived stress was assessed using the 10-item Perceived Stress Scale (PSS). • Workplace quality of life was assessed using the Professional Quality of Life Scale (ProQOL) Version 5. • Wellbeing was assessed using the Warwick Edinburgh Mental Wellbeing Scale. • Healthcare Psychological Flexibility was assessed using the Mindful Healthcare scale	Not specified	23/25 (92%) completed the intervention. 18/25 (48%) completed the questionnaire 1 month post intervention. 15/25 (60%) completed the focus group follow up.	• Findings suggest improvements in psychological flexibility and mental wellbeing between baseline and follow up, though not statistically significant. • There was minimal change in perceived stress, burnout or compassion satisfaction. • Online ACT interventions are acceptable to palliative care staff and feasible to implement on Microsoft teams. • Future studies for intervention refinement and evaluation.
5	Hulbert-Williams et al. 2021^57^	• Quality of Life assessed using the Functional Assessment of Chronic Illness Therapy (Palliative Care) scale. • Distress assessed using single item Distress Thermometer scale. • Psychological Flexibility assessed using the Comprehensive Assessment of Acceptance and Commitment Therapy (CompACT) score.	15.2% of 342 hospice referrals met eligibility criteria. Of those eligible (*n* = 52) 19.2% consented.	5/10 (50%) participants completed the intervention.	• Findings suggest intervention acceptability and perceived effectiveness. • Improvements noted in outcomes, but not statistically significant. • Future studies needed to recruit participants with non-cancer diagnoses, and earlier in the illness trajectory.
6	Johnson 2014^46^	• Prolonged Grief assessed using the PG-13 scale. • Symptom Interference measured using the Brief Symptom Inventory scale. • Acceptance assessed using the AAQ-II. • Valued living assessing using the Bulls Eye Instrument for Valued living.	Not specified.	Only 2 participants recruited for this study. Both retained.	• Both participants (*n* = 2) showed improvements in prolonged grief disorder, general clinical symptoms, value directed behaviour, gains in acceptance.
7	Kohle et al 2017^68^	Partners were asked about experiences regarding: a.Web based interventions in general, b. psycho-education (lessons and psychological exercises),c. mindfulness exercises, d. peer support, e. practical information, tips and references. For each of the topics, partners were asked what they appreciated, learnt and any suggestions for improvements.	30/52 (58%) indicated they would be willing to participate (20 were randomly selected).	14/20 (70%) participants completed an interview evaluating the intervention.	• Partners of cancer patients appreciated the Web-based self-help intervention. • Ambivalent feelings towards the peer-support aspect and counsellor feedback provided as part of the intervention. • Participants reported that the intervention helped them to cope with negative emotions, thoughts, to practice self-kindness; to clarify values based on difficult recent experiences; and gain insight and acknowledgement. • A web-based intervention based on Acceptance and Commitment Therapy and Self-Compassion may have value in supporting caregivers of patients with cancer.
8	Mosher et al. 2022^55^	• Feasibility and acceptability was assessed using the enrolment and attrition rates and participants opinions. • Patient fatigue interference was assessed using the 7-item Fatigue Interference subscale of the FSI. • Caregiver burden was assessed using the 12-item short form of the Zarit Burden Interview. • Patient sleep interference was assessed using the 8-item PROMIS sleep related impairment measure. • Patient and caregiver psychological flexibility was assessed using AAQ-II questionnaire. • Patient quality of life was assessed using the 15-item McGill Quality of Life Questionnaire-Revised. • Caregiver quality of life was assessed with the 10-item PROMIS measure of global health. • Patient and caregiver engagement in daily activities was assessed using the 6-item PROMIS measure of participation in social roles and activities and 5-item Progress subscale of Valuing questionnaire.	42/348 (12%) of gastrointestinal cancer patients approached consented. 42/77 (55%) of caregivers approached consented. Overall, eligibility screening rate was 54%.	64/80 (80%) completed all six sessions. 65/80 (81%) were retained at 2 weeks post-intervention. 58/80 (73%) were retained at 3 months post intervention.	• Group differences in outcomes were not significant. • Intervention group patients showed a moderate decline in fatigue interference compared to control group which showed no change. • Intervention group caregivers showed a medium decline in burden at 2 weeks that was not sustained at 3 months, whereas the control group showed no change. • ACT for caregivers and patients showed improvements in most quality of life outcomes across follow ups compared to the control group.
9	Mosher et al. 2019^48^	Patient Symptom Interference with function assessed by: • MD Anderson Symptom Inventory – 6-item global symptom interference subscale • Fatigue Symptom Inventory • PROMIS 4-item subscale for pain interference • PROMIS single item on task avoidance relating to dyspnoea Distress assessed using PROMIS anxiety and depression measure and the single item Distress Thermometer. Severity of patient physical symptoms assessed by: • PROMIS fatigue measure • PROMIS sleep disturbance measure • PROMIS pain intensity measure. Patient Breathlessness assessed using the Memorial Symptom Assessment Scale. Patient and caregiver acceptance was measured using PEACE questionnaire.	86/ 331 (26%) of lung cancer patients approached consented. 55/ 86 (64%) caregivers approached consented. Overall eligibility screening rate was 51%. 50/55 (91%) dyads consented and began intervention.	39/50 (78%) dyads completed all six sessions. 38/50 (78%) were retained at 6-week follow up. Overall retention was 76% at 6-week post-intervention.	• No significant difference in outcomes for the Acceptance and Commitment Therapy intervention compared to the control education/support condition. • Both groups showed a small, significant decrease in struggle with the illness. • Telephone-based Acceptance and Commitment Therapy was feasible for many advanced lung cancer patients and caregivers, but may not substantially reduce symptom interference and distress.
10	Mosher et al. 2018^49^	Patient Symptom Interference assessed using: • MD Anderson Symptom Inventory subscale • PROMIS scale for pain interference • Fatigue Symptom Inventory • PROMIS scale for sleep-related impairment. Severity of physical symptoms was assessed using: • PROMIS pain intensity measure • PROMIS short-form fatigue measure • PROMIS short-form sleep disturbance measure. Depressive and anxiety symptoms assessed with the four-item PROMIS short-form depression measure and four-item PROMIS short-form anxiety measure respectively.	50/158 (32%) approached consented. 47/50 (94%) who consented began the intervention.	39/47 (83%) completed the 8-week follow up. 37/47 (79%) completed the 12-week follow-up.	• No difference inpatient symptom interference or symptom severity outcomes for the Acceptance and Commitment Therapy and control groups. • Decreases in symptom interference for the Acceptance and Commitment Therapy group at 8 and 12 weeks compared to baseline. • Both Acceptance and Commitment Therapy and control group showed reductions in depressive symptoms at 12 weeks post-baseline.
11	Muscara et al. 2020^66^	• Posttraumatic stress symptoms (PTSS) assessed using Posttraumatic Stress Disorder Checklist V5. • Depression, anxiety, and stress were assessed using the Depression, Anxiety and Stress Scale-21. • Adjustment and experience of illness were assessed using Parent Experience of Child Illness Scale. • Ability to care for child with chronic condition was assessed using the Family Management Measure. • Acceptance measured using AAQ-II questionnaire. • Mindfulness assessed using Five Facet Mindfulness Questionnaire Short Form. • Value-based living assessed using Valuing Questionnaire. Psychological Flexibility assessed using Parent Psychological Flexibility Questionnaire.	313/1232 (25%) of those assessed for eligibility were randomised.	81/313 (26%) completed the post intervention questionnaire: 44/161 (27%) in the wait list control group retained 37/152 (24%) in the intervention group were retained.	• Intervention group demonstrated significantly greater improvements in PTSS compared with the waiting list group. • Statistically significant improvements in experience and impact of the illness on parents, and in emotional resources in the intervention compared with the control group. • Online group Acceptance and Commitment Therapy can improve post-traumatic stress symptoms in parents of children with life-threatening illness.
12	Plumb Vilardaga, et al. 2020^52^	• Feasibility assessed using the recruitment rate, accrual and retention. • Acceptability assessed via treatment engagement and client satisfaction (Client Satisfaction Questionnaire). • Pain assessed using Brief Pain Inventory Scale. • Pain Interference was assessed using Pain Disability Index. • Fatigue assessed using by Patient Reported Outcomes Measurement Information System Fatigue Short Form. • Psychological Distress assessed using the Hospital Anxiety and Depression Scale. • Mindfulness and psychological acceptance were assessed using AAQ-II. • Psychological wellbeing was assessed using Bull’s Eye Value Survey.	32/122 (26%) of participants responded to the recruitment letter. 24/32 (75%) consented to participation following receipt of study information.	21/24 (88%) of participants completed the study.	• High levels of satisfaction with the intervention, with 87% stating they would return for a refresher course and describing it as helpful or very helpful. • Engagement was high with 95% (21/22) completing all sessions. • Improvements were noted in secondary outcomes in particular value-based action and health, but the study was not powered to detect statistically significant improvements. • Intervention was feasible and acceptable and increased access to psychological support for community-based patients with advanced cancer.
13	Rost et al. 2012^53^	• Acceptance assessed using the COPE Acceptance subscale. • Mental Disengagement assessed using the COPE Mental Disengagement subscale. • Depression assessed using Beck Depression Inventory II. • Anxiety assessed using the Beck Anxiety Inventory. • Distress assessed using the Profile of Mood States. • Emotional Control assessed using The Courtland Emotional Control Scale. • Thought suppression assessed using The White Bear through Suppression Inventory. • Coping assessed using the COPE. • Quality of life assessed using the Functional Assessment of Cancer Therapy.	47/57 (82%) approached consented and began trial.	31/47 (66%) completed the intervention.	• Acceptance and Commitment Therapy group showed significantly greater decreases in psychological distress and increases in quality of life compared to the Treatment as Usual group over time. • Effects of treatment were mediated by cognitive avoidance. • Mental disengagement decreased significantly in Acceptance and Commitment Therapy group over time but increased in the TAU group. • Significant improvements in depression and anxiety in both Acceptance and Commitment Therapy and TAU group, but the improvements on both outcomes were significantly greater in the Acceptance and Commitment Therapy group.
14	Serfaty et al. 2019^60^	• Feasibility of recruitment and retention. • Attitudes to engagement assessed using the Acceptable feasibility criterion. • Functioning assessed using the FACT-G. • Psychological Distress assessed using the Kessler Psychological Distress Scale. • Physical functioning assessed using walking test and sit to stand test. • Acceptance assessed using AAQ-II questionnaire. • Living according to one’s values was assessed using the Valued Living questionnaire.	43/70 (61%) approached were assessed for eligibility. 42/54 (78%) target participants were recruited.	24/42 (57%) completed follow up at 1.5 month. 18/42 (43%) completed follow up at 3 months. 15/42 (36%) completed follow up at 6 months.	• 78% of target recruited. • 43% retained at 3 months, lower than target (60%). Reasons for attrition included death, deteriorating health and loss to follow-up. One person felt the therapy was unsuitable. No significant differences in attrition across groups. • Participants engaged well with therapy and reported satisfaction with the intervention they received. • Acceptance and Commitment Therapy in the palliative population is acceptable but researchers need to reduce the burden of research on patients to optimise engagement.
15	Wells-Di Gregorio et al. 2018^54^	• Sleep difficulties assessed using the National sleep foundation sleep diary and Insomnia severity index. • Worry assessed using the Penn State Worry Questionnaire. • Intolerance of uncertainty assessed using the Intolerance of Uncertainty Scale. • Day time sleepiness assessed using Epworth Sleepiness Scale. • Anxiety assessed using the State-Trait Anxiety Inventory scale. • Depressive sleepiness assessed using the Centre for Epidemiological Studies Depression Scale. • Fatigue assessed using Fatigue Symptom Inventory. • Distress assessed using the James Supportive Care Screening. Hyperarousal assessed using Impact of Event Scale-Revised.	30/1073 (3%) assessed for eligibility were randomised. • 860/1073 (80%) did not meet the eligibility criteria • 183/1073 (17%) declined to participate 28/30 (93%) began the intervention.	21/28 (75%) completed the intervention.	• CBT-ACT group showed significant improvements in sleep efficiency, sleep latency, insomnia and worry compared with the waitlist control group. • Significant improvements on sleep related outcomes, hyper arousal, worry and depression were noted for the total sample (both groups combined) between baseline and 6-week follow-up.

AAQ-II: Acceptance and Action Questionnaire II.

PG: Prolonged Grief.

PROMIS: Patient Reported Outcomes Measurement Information System.

TAU: Treatment as Usual.

#### Settings

Four of the 15 intervention studies (27%) described interventions in hospitals^16,53,62,63^, eight (53%) described interventions in home settings.^46,48,49,52,55,61,66,68^ One intervention was delivered in both the hospital and community (7%)^54^ and one was hospice-based (7%).^57^ One paper described an intervention delivered in a hospice, therapist’s clinic or home (7%).^60^ See Supplemental File: Figure 5.

#### Intervention mode of delivery

Most interventions evaluated were individual focused (*n* = 8; 53%)^46,49,52–54,60,63,68^; three were group^57,62,66^, four were mixed (see Supplemental File: Figure 6).^16,48,55,61^ Interventions were most commonly delivered in person (*n* = 5, 33%),^46,53,57,60,62^ or by telephone (*n* = 5; 33%)^48,49,52,55,68^ (see Supplemental File: Figure 7).

#### Types of professionals delivering acceptance and commitment therapy interventions

Nearly half of the evaluated interventions (7/15; 47%) were delivered by specialists such as clinical psychologists.^16,52–54,57,60,61,63,66,68^ A further three (20%) were delivered by social workers,^48,49,55^, three (20%) were delivered by clinical psychology students.^53,63,68^ One was delivered by an MSc student and supervised by a clinical psychologist (7%)^46^ (see Supplemental File: Table 2).

#### Intervention content

All interventions incorporated the six core Acceptance and Commitment Therapy processes. Four combined Acceptance and Commitment Therapy with another intervention – traditional CBT,^54^ Coping Skills Training,^52^ Problem-solving Skills Training.^62^ The studies used a variety of media, with five studies providing homework in the form of audiobooks,^52,66^ online modules,^16,61^ DVDs^54^ and booklets^52,63,66^ (Table 3).

For treatment integrity, four of the intervention studies (27%) assessed intervention fidelity with developed checklists to ensure adherence to the treatment protocol^48,49,55,66^, four other studies carried out informal fidelity checks.^52,54,57,61^ Three of the papers stated that in future research they would develop fidelity checklists.^46,60,61^

#### Duration and frequency of interventions

Total duration of the interventions varied from 15 min to 12 h (Table 3). The longest intervention consisted of 12 weeks of 1 h in-person sessions delivered in hospital to people with advanced cancer.^53^ The shortest duration was a one-off individual phone call (15–60 min) for informal caregivers.^68^ Intervention sessions ranged from 15 to 120 min: in-person based sessions (60–90 min) and technology-based sessions (45–120 min). Group-based sessions lasted longer than the individual-based sessions. Two of the papers did not specify the duration of their intervention sessions (13%).^46,63^

The frequency of the interventions ranged from one-off^63,68^ to 12 sessions.^53^ The 12-session study adopted an individual in-person mode of delivery.^53^ Two thirds of the papers delivered weekly sessions (*n* = 10)^46,48,49,54,55,57,60–62,66^ with three delivering booster sessions a month after the last session.^57,62,66^

#### Data collection points during interventions

Most intervention studies (*n* = 14/15) collected outcome data pre and post intervention.^16,46,48,49,52–55,57,60–63,66^ Ten of these further assessed their outcomes 1–12 months after the intervention was delivered.^16,46,48,49,53–55,61–63^

#### Outcome measures reported in intervention studies

A range of physical and psychological outcome measures were assessed (see Supplemental File: Figure 8/9). Psychological flexibility was assessed in nearly all papers (*n* = 12; 80%),^46,48,52,53,55,57,60–63,66,68^, four of these used the AAQ-II to assess psychological flexibility.^55,60,63,66^ Other key outcomes assessed included anxiety (*n* = 6),^16,52–54,63,66^ depression (*n* = 6),^16,52–54,63,66^ distress (*n* = 7),^48,52–54,57,60,62^ valued living (*n* = 7)^46,52,55,60,63,66,68^ and pain interference (*n* = 5).^16,46,48,49,52^

#### Feasibility and acceptability of acceptance and commitment therapy

Six of the 15 intervention studies examined feasibility and acceptability as one of their stated outcomes.^16,52,55,60,61,63^ Enrolment rates varied from 19-82%. The studies reporting the lowest levels of recruitment involved participants recently referred to a hospice service in the UK (19% of those eligible consented),^57^ and an intervention involving parents with elevated stress symptoms with a child admitted to hospital for a life-threatening illness or injury (25% consented). Recruitment was highest (82%) in a study involving patients with Stage III or Stage IV ovarian cancer, in which eligible patients were informed about the study during their routine clinic, and the intervention was scheduled to take place alongside their routine appointments to minimise travel and time requirements.^53^ Across all interventions studies, retention rates ranged from 26-88%. Retention was highest (88%) for a study which used an individual telephone based intervention with sessions lasting 45 min, removing barriers of in-person intervention such as illness and travel.^52^ It lowest for a study involving six-session online group intervention for in parents of children with a life-limiting illness.^62^ Detailed data on enrolment and retention data can be found organised by participant population in Table 4 in Supplemental File 1.

Twelve studies (80%) reported patient satisfaction rates with their interventions^16,49,52–54,57,60–63,66,68^; and interventions were generally acceptable to participants in all studies. Detailed data on satisfaction rates organised into population group in Table 5 in the Supplemental File 1.

**Table 5.** Details of observational studies (*N* = 11).

	AuthorS (YR)	Title	Aims/purpose	Study Method	Outcomes and details of these (e.g. how measured)	Key findings that relate to the scoping review questions
1	Al-Hammouri et al, 2020^69^	Exploring the potential of acceptance and commitment therapy model in self-care behaviour in persons with heart failure	To examine the interaction among cognitive fusion, mindfulness and committed action on the self-care behaviour in person with heart failure as guided by the acceptance and commitment therapy model.	Quantitative Questionnaires Patient *N* = 165 patients with a diagnosis of heart failure.	• Self-care behaviour was assessed using the SCHFI-V6 scale. • Cognitive Fusion was assessed using CFQ . • Mindfulness was assessed using the Mindful Attention Awareness Scale. • Committed action was assessed using the CAQ-8.	• Significant positive associations were shown between the Acceptance and Commitment Therapy model and self-care behaviour, mindfulness and committed action. No effects were shown for cognitive fusion.
2	Davis et al, 2016^64^	Prediction of individual differences in adjustment to loss: Acceptance and valued-living as critical appraisal and coping strengths	To explore the relationships between acceptance and valued living with grief and fear of death in a sample of bereaved university students.	Quantitative Questionnaires Bereaved *N* = 97 bereaved students who have lost family member or friend in last 2 years).	• Grief: Assessed using PG-13 questionnaire. • Acceptance: Assessed using the Acceptance and Action Questionnaire-II. • Valued Living: Assessed using the Valued Living Questionnaire. • Death Attitudes: Assessed using Multi-dimensional Orientation Towards During and Death Inventory. • Communication Avoidance: Assessed by the Expressiveness subscale of the 12 item Family Relationship Index.	• Acceptance was positively associated with death acceptance and negatively associated with fear of death and communication avoidance. • Valued living was associated with acceptance and fear of death outcomes but not with communication avoidance. • Results provided preliminary support for the importance of acceptance and valued living in predicting individual differences in adjustment to loss and have implications for supporting the bereaved.
3	Davis et al 2017^65^	Is Higher Acceptance Associated with Less Anticipatory Grief Among Patients in Palliative Care?	To assess the relationships between acceptance, anticipatory grief, anxiety, and depression among patients in palliative care.	Quantitative Questionnaires Patient *N* = 73 patients with a life limiting illness.	• Acceptance: Assessed using AAQ-II questionnaire. • Anticipatory Grief: assessed using the PG-12 scale. • Anxiety and Depression: Hospital and Anxiety Depression Scale.	• Anxiety and depression were significant predictors of anticipatory grief. • Higher acceptance was associated with lower anticipatory grief in patients. • Higher acceptance was associated with lower anxiety and depression. • It provides sufficient support for future research examining the effect- liveness of acceptance-based (e.g., Acceptance and Commitment Therapy) interventions for patients with problematic levels of anticipatory grief.
4	Edwards 2021^45^	Exploring the Moderating Effects of Psychological Flexibility on Compassion, Satisfaction and Fatigue in the Context of End-of-life Care Within a Hospital Setting	To examine how factors such as education, years of experience, and exposure to death and dying may be useful predictors for compassion satisfaction, compassion fatigue, and competency in delivering End of Life care.	Quantitative and Qualitative Questionnaires. Bereaved and Formal caregivers *N* = 57 nursing staff members.	• Experience with End of life was assessed. • Support Usage was assessed with descriptions of how staff manage their distress. • Compassion fatigue was assessed using the Professional Quality of Life scale. • Comfort and competency was assessed using the End-of-Life Professional Caregiver Survey. • Acceptance was assessed using the AAQ-II questionnaire. • Open ended questions were used to assess the impact of losing a patient.	• Years of experience was the only significant predictor of compassion fatigue, with more years of experience being predictive of lower levels of compassion fatigue. • A moderate relationship between psychological flexibility and exposure to death and dying and compassion satisfaction/fatigue and competency in delivering end-of-life care. • Acceptance was negatively correlated with compassion satisfaction and positively correlated with compassion fatigue scores.
5	Fisher et al 2021^56^	The Experience of palliative care professionals and their responses to work related stress: A Qualitative Study	To understand and describe the experiences of PCPs (palliative care professionals) and to explore the helpful and unhelpful responses to work related stress they employ.	Qualitative Interview Staff *N* = 9 palliative care professionals.	Analysis was based on framework method: • Sources of Meaning and Purpose • Sources of Stress • Personal Impact • Unhelpful Reponses • Helpful Responses.	• Five themes were identified (1) Sources of Meaning and Purpose (making a difference, personal growth), (2) Sources of Stress (emotional challenges, patient family dynamics, work environment factors, public perception, uncontrollability of symptoms), (3) Personal Impact (life engagement, perceptions of death), (4) Unhelpful Responses (self-doubt, emotional suppression, rumination, overidentifying, lack of self-care), and (5) Helpful Responses (acceptance, being present, perspective taking, being able to switch off, social support, active self-care.
6	Low et al. 2012^58^	The role of acceptance in rehabilitation in life-threatening illness	To explore the relationship between acceptance and psychological and physical status.	Quantitative questionnaire and physical function test. Patient *N* = 101 patients participated (42 metastatic disease, 14 non cancer, 16 first recurrence, 29 first remission).	• Acceptance: Assessed using AAQ-II questionnaire. • Psychological Status: Assessed using the Kessler 10 score. • Physical Function: assessed using a 1-min time sit to stand test.	• Statistically significant negative association between acceptance and psychological morbidity and a positive association between acceptance and sit to stand and distance walked.
7	Martin et al 2022^67^	The role of psychological flexibility in palliative care.	To test the applicability of the Psychological Flexibility Framework in accounting for variability in four palliative patient outcomes (death attitudes, distress, pain and QoL) and short-term stability of these variables in the PC context.	Quantitative questionnaire. Patient *N* = 81 patients with a prognosis of 12 months or less.	• Psychological Flexibility was assessed using Comprehensive Assessment of Acceptance and Commitment Therapy (CompACT) score. • QoL was assessed using 4-item McGill Quality of Life Questionnaire-Revised (MQOL-R). In addition, it was assessed via a secondary measure by carers using the same questionnaire. • Distress was assessed using the 14-item Hospital Anxiety and Depression Scale. • Death Attitudes was assessed using the 32-item Death Attitude Profile-Revised. • Physical Pain was assessed using the 9-item Brief Pain Inventory.	• Results showed that PF remained stable over the 1-month study interval in accord with stable palliative patient outcomes. • Total psychological flexibility emerged as a significant predictor of palliative patient quality of life, distress and death attitudes. • Unexpected findings: Higher valued action measure was associated with worse pain outcomes. • Behavioural awareness measure was not a predictor of any patient outcomes.
8	Mosher et al 2021^47^	Symptom experiences in advanced cancer: Relationships to acceptance and commitment therapy constructs	To explore the relationship between acceptance and commitment therapy (ACT) constructs and symptom-based subgroups of advanced cancer patients.	Quantitative questionnaire. Patient *N* = 201 patients with advanced breast, gastrointestinal, lung, and prostate cancer.	• Psychological Inflexibility was assessed using the AAQ-II questionnaire. • Cognitive Fusion was assessed with Cognitive Fusion Questionnaire. • Values-based living was assessed using the Valuing Questionnaire. • Peaceful acceptance was assessed using the 7-item Peaceful Acceptance subscale of the Peace, Equanimity, and Acceptance in the Cancer Experience (PEACE) measure. • Mindfulness was assessed using the Awareness, Nonjudging, and Nonreactivity subscales of the Five Facet Mindful- ness Questionnaire-Short Form (FFMQ-SF). • Activity Engagement was assessed using PROMIS^a^ measure of ability to participate in roles and activities, including leisure, social roles, and work (including housework).	• Advanced cancer patients show heterogeneous symptom profiles, and even mild to moderate symptom levels are related to greater withdrawal from personally meaningful activities and less acceptance of cancer and internal experiences (e.g., symptoms, thoughts, feelings).
9	Mosher et al 2017^50^	Symptom experiences in metastatic breast cancer patients: Relationships to activity engagement, value-based living, and psychological inflexibility	To examine the symptom-based subgroups of metastatic breast cancer patients and the extent to which they differed across key constructs of acceptance and commitment therapy.	Quantitative questionnaire. Patient *N* = 80 patients with MBC (stage IV).	• Physical symptoms were assessed using PROMIS measure and Memorial Symptom Assessment Scale. • Engagement in roles and activities was assessed using the PROMIS measure. • Psychological Inflexibility was assessed using AAQ-II . • Valued base living was assessed using VQ questionnaire.	• Women with metastatic breast cancer (MBC) show heterogeneity in their symptom profiles, and those with higher symptom burden were more likely to disengage from valued activities and avoid unwanted experiences (e.g., thoughts, feelings, and bodily sensations). • Decreased psychological flexibility was associated with decreased valued living.
10	Murrell et al 2018^51^	Psychological Flexibility and Resilience in Parentally Bereaved College Students	To explore the relationships among bereavement related distress, experiential avoidance, values, and resiliency in parentally bereaved college students.	Quantitative questionnaire Bereaved *N* = 63 students studying at university and who have experienced a death of a parent before the age of 18.	• Valued living was assessed via the Valued Living Questionnaire. • Experiential avoidance was measure using the Avoidance and Fusion Questionnaire for Youth. • Coping Ability response to stress was assessed using Connor-Davidson Resiliency Scale. • Bereavement experience was assessed using the Bereavement Experience Questionnaire.	• Experiential avoidance and low importance of values were correlated with bereavement difficulties. • No significant relationship between measures of experiential avoidance and values.
11	Walker 2013^59^	The Experience of Therapist and Bereaved Clients of using an Acceptance and Commitment Therapy approach to grief	To examine Acceptance and Commitment Therapy as a treatment approach to grief.	Qualitative interview. Staff *N* = 6 Therapists and 2 clients.	Analysis was based on respective superordinate and subordinate themes: Therapists • Facing Grief with Acceptance and Commitment Therapy • Factors shaping application • The purpose of theoretical knowledge. Clients • The presence of undesirable thoughts and feelings • Client perceptions.	• Acceptance and Commitment Therapy components were found to be useful by both therapists and clients in their practice and life. • Findings indicated that therapists were using core concepts of Acceptance and Commitment Therapy in their care for coping strategies for strong emotions experienced by clients, acceptance and positive adjustment following bereavement.

a PROMIS: Patient Reported Outcomes Measurement Information System.

#### Effectiveness of acceptance and commitment therapy interventions

Intervention studies reported on a wide range of outcome measures, broadly suggesting that Acceptance and Commitment Therapy results in improvements in a range of outcomes, though some improvements were not statistically significant (Supplemental File: Table 3). No studies identified negative or adverse impacts of Acceptance and Commitment Therapy.

##### Psychological flexibility

Nine studies reported improvements in psychological flexibility, though only two RCTs reported statistically significant improvements.^53,66^ The remaining studies showed improvements that were not significant: pre/post study designs (*n* = 5)^46,52,57,61,62^ and RCTs (*n* = 3).^48,55,63^ The qualitative study reported comments of participants showing increased psychological flexibility.^68^

##### Anxiety

All of the studies which measured anxiety showed improvements (*n* = 6), although only two RCTs reported statistically significant improvements.^53,54^ The other studies showed improvements that were not significant, pre/post designs (*n* = 2)^16,52^ and RCTs (n=2).^63,66^

##### Depression

All of the studies (*n* = 6) which measured depression showed improvements, only two RCTs showed statistically significant improvements.^53,54^ Two pre/post studies^16,52^, two RCTs^63,66^ showed improvements that were not significant.

##### Distress

Six studies showed improvements in distress, with three RCTs showing statistically significant improvements^53,54,60^, three pre/post studies showing not statistically significant improvements.^52,57,62^

##### Valued living

Six studies showed improvements in valued living post intervention,^46,52,60,63,66^ although only one RCT showed statistically significant improvements.^66^ Two pre/post studies^46,52^, two RCTs showed non statistically significant improvements.^60,63^ The qualitative study showed that a number of participants post intervention had an increased awareness of values in life and were living in accordance with personal values.^68^ One study showed no improvements in valued living post intervention.^55^

##### Pain interference

Only two of five of the studies examining pain interference showed improvements (40%), one pre/post study^52^ and one RCT^16^ showed improvements that were not significant. Three studies found no change; two RCTs^48,49^, one pre/post study.^46^

##### Fatigue interference

Four studies reported improvements in fatigue interference, of these only one RCT showed statistically significant improvements.^49^ The remaining three studies, two RCTs^54,55^, one pre/post design^52^ described improvements that were not statistically significant.

##### Sleep interference

Three studies showed improvement in sleep interference, two RCTs reporting statistically significant improvements.^49,54^ One study did not show any improvements with sleep interference post intervention.^49^

##### Quality of life

Three studies showed improvements in quality of life add^53,55,57^, one study reported significant improvements.^53^ and one pre/post study^57^ and one RCT^55^ described improvements that were not significant. One pre/post study showed minimal change to quality of life post intervention.^61^

##### Other outcome measures

Two studies described improvements in grief and anticipatory grief post intervention,^46,63^ though these were not statistically significant, and one was a PhD study involving only two participants. One pre/post study reported improvements that were not significant in mindfulness post intervention, but this was not statistically significant.^62^

There was heterogeneity in outcome measures throughout the studies, many outcome measures were only reported in one study (Supplemental File: Figure 2). Four studies described statistically significant improvements in outcomes post intervention; emotional control,^53^ thought suppression,^53^ post-traumatic stress symptoms^66^ and worry.^54^

Qualitative evidence exploring the effectiveness of Acceptance and Commitment Therapy showed that caregivers found the intervention helped them to cope with negative emotions and thoughts.^68^ Also, the intervention helped the caregivers become more aware of their personal values.^68^ The caregivers expressed ambivalent feelings towards the peer support and content of feedback from the counsellor.

#### Findings from observational studies (n=11)

Eleven observational papers were identified.^45,47,50,51,56,58,59,64,65,67,69^ The most frequently assessed outcomes were acceptance (*n* = 6)^45,47,50,58,64,65^ and valued living^47,50,51,64,67,69^ both assessed in six studies (see Table 5).

##### Acceptance

Several papers found that higher acceptance was associated with statistically significant improvements in anticipatory grief,^64,65^ anxiety,^65^ depression,^65^ physical function^58^ and low levels of burnout and stress.^56^ There was also an association between higher acceptance scores and greater levels of work engagement, job satisfaction and wellbeing in staff.^56^ A cross-sectional questionnaire study of people approaching end of life in an inpatient palliative care unit reported that acceptance was a significant predictor of reduced anticipatory grief over and above depression and anxiety, suggesting that acceptance-based interventions could play an important role in ameliorating suffering relating to anticipatory grief.^65^ A doctoral thesis reported that therapists used acceptance and commitment therapy and positive adjustment in their practice for patients following bereavement.^59^

##### Valued living

Statistically significant interactions were found between mindfulness, committed action and self-care behaviours.^69^ One study found that avoidance and low importance of values in bereaved populations was correlated with bereavement difficulties.^51^ Unexpectedly, in one study higher scores of valued action were significantly correlated with higher scores of physical pain and death attitudes.^67^

##### Grief and anticipatory grief

Higher acceptance was statistically significantly associated with lower anticipatory grief.^65^ Also, increased acceptance and valued living was statistically significantly associated with improvements in adjustment to loss amongst bereaved university students.^64^

## Discussion

This is the first review to comprehensively map the evidence on Acceptance and Commitment Therapy for people with an advanced progressive illness and their formal and informal caregivers. To date, most research has been conducted in the U.S, most studies have involved patients, and most interventions have been community-based, and delivered in-person or via telephone. Our findings suggest that Acceptance and Commitment Therapy is acceptable to people with an advanced progressive illness and those involved in their care. Across all studies, Acceptance and Commitment Therapy interventions were feasible to evaluate, at least in the short term. Nearly all intervention studies described improvements in distress, anxiety, depression, and psychological flexibility, though not all improvements were statistically significant, often due to underpowered studies. No negative impacts of Acceptance and Commitment Therapy were reported in any intervention study. Observational studies described significant associations between core Acceptance and Commitment Therapy processes such as acceptance and key psychological outcomes including adjustment to loss.

Overall, recruitment to Acceptance and Commitment Therapy intervention studies generally appeared feasible, with most studies reporting recruitment rates of at least one-third of those eligible. Challenges associated with recruitment and retention of participants in palliative care studies are widely recognised, with factors such as deteriorating health, feelings of being overwhelmed, and fatigue, all contributing to low levels of recruitment and retention.^71,72^ Gatekeeping is also an issue with ‘fear of burdening vulnerable patients’ the most reported reason for gatekeeping among healthcare professionals.^73^ In this scoping review, recruitment was lowest in an intervention study recruiting participants during the period in which they had just been referred for hospice care^57^ – a time during which patients can feel heightened levels of distress, while professionals may be more likely to gatekeep as they focus on clinical care. In contrast, recruitment was highest where the intervention took place alongside routine appointments, minimising the need to travel and reducing overall research burden.^53^ Strategies to improve recruitment include having research staff on-site, limiting burden by running intervention alongside routine care, offering flexibility around location and timing of participation, engaging patient representatives in the design of recruitment strategies, and working closely with healthcare professionals and leaders to promote research engagement.^71–73^

This review provides strong evidence that Acceptance and Commitment Therapy is acceptable in palliative care settings. In most intervention studies, attrition was low during intervention delivery, with most studies reporting retention rates of 75% or higher. Evidence regarding acceptability resonates with findings from a recent UK national survey which reported that 41% of hospice psychological support teams use Acceptance and Commitment Therapy to support patients and families.^41^ This suggests that even though evidence is still emerging, practitioners find Acceptance and Commitment Therapy a valuable psychological approach in palliative care. This could be due to its logical fit with the needs of patients with advanced progressive illness, or the strength of evidence for Acceptance and Commitment Therapy in other health populations, or it could be due to the finding that training in Acceptance and Commitment Therapy has benefits for both therapeutic skills and for personal wellbeing.^74–76^

We found preliminary evidence that Acceptance and Commitment Therapy is effective in improving key psychological outcomes for patients, carers and palliative care staff. Following Acceptance and Commitment Therapy, participants in most studies reported improvements in psychological flexibility,^46,52,53,55,57,61–63,66,68^ anxiety,^16,52–54,63,66^ and depression.^16,52–54,63,66^ Although preliminary, these findings reflect the benefits of Acceptance and Commitment Therapy reported for other populations, including people with mental health problems and chronic pain.^26,77^ Evidence for benefits of Acceptance and Commitment Therapy on physical outcomes such as fatigue, sleep and pain was limited. Weak effects for pain interference were found, with half of the papers assessing pain showed no change post intervention.^16,48,49^ Findings in palliative care populations broadly align with findings from a review of Acceptance and Commitment Therapy for chronic pain, which found significant effects of Acceptance and Commitment Therapy on pain acceptance, but no impact on pain severity.^77^ Overall, given the small sample sizes, focus on feasibility and acceptability over efficacy and effectiveness in many studies, and use of pre-post designs, further research examining the effectiveness of Acceptance and Commitment Therapy on key psychological and physical outcomes in palliative care populations is warranted.

There is existing evidence that acceptance is an important element for a good death and a facilitator in the provision of end-of-life care.^78^ Findings from observational studies in this scoping review supports the view that acceptance is a key process in palliative care. Acceptance was associated with improved anxiety,^65^ depression,^65^ death acceptance,^64^ lower anticipatory grief^64,65^ and mobility.^58^ Greater acceptance was also linked with higher levels of work engagement, job satisfaction and wellbeing in palliative care staff.^56^ In Acceptance and Commitment Therapy, acceptance describes a willingness to open-up and make room for difficult thoughts and emotions, when this helps the person accomplish something they care about. It is viewed as an active choice that involves decreasing the effort that one exerts to control or regulate inner experience, and actively experience things as they are.^52–54,57,60,62^ For instance, acceptance may involve a willingness to experience difficult thoughts and feelings associated with an advanced progressive illness in the service of communicating preferences regarding future care and plans. This contrasts with experiential avoidance which might involve attempts to reduce or avoid unwanted inner experiences when doing so has a negative impact on functioning, or may get in the way of doing what is important and most valued. Promoting acceptance amongst people with an advanced progressive illness, and their carers, may support better communication and clarity around future care preferences. Recent evidence from a feasibility study points to the acceptability of Acceptance and Commitment Therapy to address advance care planning needs of depressed and anxious adults with advanced cancer.^16^ Further research on this relationship is warranted

In this scoping review, short or flexibly delivered interventions resulted in greater participant retention.^16,48,49^ Brief psychosocial interventions have been found to be effective in improving clinically relevant outcomes for people receiving palliative care.^38,79^ Illness stage is also an important consideration, with shorter, flexible intervention formats being essential for those using hospice services and closer to the end of life. We identified two hospice-based interventions studies in this review. In one study, 4 of 10 participants recruited died during the study^57^ in the other, only 8 of 20 (40%) allocated to the 8-week intervention condition completed the 1.5 month follow-up due to death, feeling too unwell, feeling overburdened and related reasons.^60^ Given deteriorating physical health, earlier intervention is important for people with an advanced progressive illness, and where this is not feasible, brief and flexible interventions, which do not overburden the person, need to be prioritised.

## Strengths and limitations

This is the first comprehensive review focused on Acceptance and Commitment Therapy in palliative care settings, synthesising evidence for people with any advanced progressive illness, their caregivers, and staff involved in their care. This review, which draws on data from 26 research studies, extends the findings from a systematic review in this area, which summarised findings from six studies involving people with advanced cancer.^38^ Given the rapid growth in Acceptance and Commitment Therapy as an effective psychological intervention for people with a range of conditions,^29^ this review is timely.

A number of limitations are noted. First, a high degree of heterogeneity in relation to study populations, aims, intervention designs and format made some comparisons difficult. Secondly, the lack of control group in most studies limited the strength of evidence, such that only preliminary evidence on effectiveness could be described. Thirdly, as most intervention studies focused on people with advanced cancer, conclusions regarding the use of Acceptance and Commitment Therapy for other populations was limited. As this was a scoping review, we focused on mapping the broad field rather than critically appraising papers, and quality appraisal was not undertaken. Choices regarding study inclusion were pragmatic, and papers in languages other than English were excluded; therefore, findings may be limited in the extent to which they apply across cultures.

## Directions for future research

Of the 26 studies reviewed, only three focused on Acceptance and Commitment Therapy for bereavement support. The dearth of evidence on Acceptance and Commitment Therapy for bereavement was also recently noted in a systematic review on managing grief, which only identified two studies.^40^ Future research is needed to explore the acceptability and value of Acceptance and Commitment Therapy for people who have been bereaved. Similarly, only two studies involving palliative care staff were identified,^45,56^ however given the effectiveness of Acceptance and Commitment Therapy in promoting wellbeing in other occupational settings,^37^ further research examining Acceptance and Commitment Therapy for palliative care staff wellbeing is warranted. Most patient studies involved people with cancer, so research involving people with other advanced progressive or life-limiting illnesses is needed.

Interventions need to be scalable and cost-effective to implement, so future feasibility testing of interventions needs to incorporate preliminary economic evaluation. Similarly, models of intervention delivery which rely on highly specialist staff such as clinical psychologists for delivery may not be scalable, so research is needed to adapt interventions so that Acceptance and Commitment Therapy can be delivered by a wider range of professionals. There is now emerging evidence for the value of Acceptance and Commitment Therapy delivered online in other settings,^26^ so given the potential for online interventions to increase access to psychological support, future evaluation of online Acceptance and Commitment Therapy for palliative care is timely. Greater consideration of the acceptability and effectiveness of Acceptance and Commitment Therapy for diverse groups and cultures is also recommended.

In terms of research design, efficacy, effectiveness and implementation studies involving patients and carers need to be prioritised. Few full-scale evaluations of Acceptance and Commitment Therapy to support patient, carers and staff wellbeing in palliative care settings have been conducted, so evidence regarding effectiveness remains uncertain. Mixed method evaluation studies, with larger sample sizes and comparison conditions are needed. Hybrid effectiveness-implementation studies are also recommended to explore how delivery of the intervention can be scaled up, and delivered in health and social care contexts.^80,81^

## Implications for clinical practice

Whilst this field of research and practice is still early in its development, practitioners can be reassured that Acceptance and Commitment Therapy has a relatively robust evidence base in many related areas of practice, such as organisational stress, caregiver stress, chronic and mental ill health. This review provides evidence that Acceptance and Commitment Therapy interventions for people with advanced progressive illness are likely to be acceptable, and feasible to deliver, with preliminary evidence of efficacy. There is also evidence from pre-trial cross-sectional studies that the processes targetted in the Acceptance and Commitment Therapy model are related to wellbeing in people with advanced illness and therefore are logical intervention targets. Given the early stage of development of this field, it may be valuable for clinicians to routinely evaluate practice and identify unusual or boundary cases that could be written up as case studies to inform intervention development. Given the heterogeneity of delivery seen in this review, clinicians should be encouraged to explore and evaluate delivery methods in routine practice, in parallel with further research. In particular, training non psychologists / non psychotherapist to deliver elements of Acceptance and Commitment Therapy may be useful to address the relative scarcity of those professional groups, relative to others.

## Conclusion

Acceptance and Commitment Therapy is acceptable and feasible to deliver in palliative care settings. There is preliminary evidence that Acceptance and Commitment Therapy improves anxiety, depression and distress amongst those with advanced progressive illness, as well as their caregivers. Full scale mixed method evaluation studies involving larger samples and a comparison group are now needed to demonstrate efficacy, effectiveness and cost-effectiveness. Further intervention development and feasibility studies involving staff and bereaved carer populations are warranted.

## Supplemental Material

sj-pdf-1-pmj-10.1177_02692163231183101 – Supplemental material for Acceptance and Commitment Therapy (ACT) for people with advanced progressive illness, their caregivers and staff involved in their care: A scoping review Click here for additional data file. Supplemental material, sj-pdf-1-pmj-10.1177_02692163231183101 for Acceptance and Commitment Therapy (ACT) for people with advanced progressive illness, their caregivers and staff involved in their care: A scoping review by Tilly Gibson Watt, David Gillanders, Juliet A Spiller and Anne M Finucane in Palliative Medicine
