# Supplementary material for: Acceptance and Commitment Therapy (ACT) for people with advanced progressive illness, their caregivers and staff involved in their care: A scoping review
Source: Palliat Med. 2023 Jul 25;37(8):1100–28. doi: 10.1177/02692163231183101 (PMC10503261; doi:10.1177/02692163231183101)
Supplement: sj-pdf-1-pmj-10.1177_02692163231183101 – Supplemental material for Acceptance and Commitment Therapy (ACT) for people with advanced progressive illness, their caregivers and staff involved in their care: A scoping review [file sj-pdf-1-pmj-10.1177_02692163231183101.pdf]

Supplementary File

Table of Contents:

Content:	*Page Number*
Table 1: Medline Search Strategy	2-4
Table 2: Types of healthcare professionals delivering ACT interventions	5
Table 3: Results for most common outcome measures reported in ACT intervention papers	6-7
Table 4: Data on enrolment and retention data for intervention studies	8
Table 5: Data on satisfaction rate for intervention studies	9
Figure 1: Scatter graph illustrating country of publication for included studies (n=26)	10
Figure 2: Scatter graph illustrating years of publication for the included studies (n=26)	11
Figure 3: Stacked bar chart for study type of included studies (n=26)	12
Figure 4: Study populations for included studies (n=26)	13
Figure 5: Study settings for intervention studies (n=15)	14
Figure 6: Intervention format for intervention studies (n=15)	15
Figure 7: Mode of delivery of Acceptance and Commitment Therapy interventions (n=15)	16
Figure 8: Frequency of psychological outcome measures used in intervention studies (N=15)	17
Figure 9: Frequency of physical outcome measures	18
References	19-20

Table 1: Medline Search Strategy

1.	(acceptance and commitment therapy).mp. [mp=title, book title, abstract, original title, name of substance word, subject heading word, floating sub-heading word, keyword heading word, organism supplementary concept word, protocol supplementary concept word, rare disease supplementary concept word, unique identifier, synonyms, population supplementary concept word, anatomy supplementary concept word]
2.	ACT-based.mp. [mp=title, book title, abstract, original title, name of substance word, subject heading word, floating sub-heading word, keyword heading word, organism supplementary concept word, protocol supplementary concept word, rare disease supplementary concept word, unique identifier, synonyms, population supplementary concept word, anatomy supplementary concept word]
3.	ACT.mp. [mp=title, book title, abstract, original title, name of substance word, subject heading word, floating sub-heading word, keyword heading word, organism supplementary concept word, protocol supplementary concept word, rare disease supplementary concept word, unique identifier, synonyms, population supplementary concept word, anatomy supplementary concept word]
4.	psychological flexibility.mp. [mp=title, book title, abstract, original title, name of substance word, subject heading word, floating sub-heading word, keyword heading word, organism supplementary concept word, protocol supplementary concept word, rare disease supplementary concept word, unique identifier, synonyms, population supplementary concept word, anatomy supplementary concept word]
5.	contextual behavioural science.mp. [mp=title, book title, abstract, original title, name of substance word, subject heading word, floating sub-heading word, keyword heading word, organism supplementary concept word, protocol supplementary concept word, rare disease supplementary concept word, unique identifier, synonyms, population supplementary concept word, anatomy supplementary concept word]
6.	acceptance commitment therapy.mp. [mp=title, book title, abstract, original title, name of substance word, subject heading word, floating sub-heading word, keyword heading word, organism supplementary concept word, protocol supplementary concept word, rare disease supplementary concept word, unique identifier, synonyms, population supplementary concept word, anatomy supplementary concept word]
7.	1 or 2 or 3 or 4 or 5 or 6
8.	palliative care.mp. [mp=title, book title, abstract, original title, name of substance word, subject heading word, floating sub-heading word, keyword heading word, organism supplementary concept word, protocol supplementary concept word, rare disease supplementary concept word, unique identifier, synonyms, population supplementary concept word, anatomy supplementary concept word]
9.	last year of life.mp. [mp=title, book title, abstract, original title, name of substance word, subject heading word, floating sub-heading word, keyword heading word, organism supplementary concept word, protocol supplementary concept word, rare disease supplementary concept word, unique identifier, synonyms, population supplementary concept word, anatomy supplementary concept word]
10.	palliative.mp. [mp=title, book title, abstract, original title, name of substance word, subject heading word, floating sub-heading word, keyword heading word, organism supplementary concept word, protocol supplementary concept word, rare disease supplementary concept word, unique identifier, synonyms, population supplementary concept word, anatomy supplementary concept word]
11.	terminal care.mp. [mp=title, book title, abstract, original title, name of substance word, subject heading word, floating sub-heading word, keyword heading word, organism supplementary concept word, protocol supplementary concept word, rare disease supplementary concept word, unique identifier, synonyms, population supplementary concept word, anatomy supplementary concept word]
12.	hospice care.mp. [mp=title, book title, abstract, original title, name of substance word, subject heading word, floating sub-heading word, keyword heading word, organism supplementary concept word, protocol supplementary concept word, rare disease supplementary concept word, unique identifier, synonyms, population supplementary concept word, anatomy supplementary concept word]
13.	advance care planning.mp. [mp=title, book title, abstract, original title, name of substance word, subject heading word, floating sub-heading word, keyword heading word, organism supplementary concept word, protocol

	supplementary concept word, rare disease supplementary concept word, unique identifier, synonyms, population supplementary concept word, anatomy supplementary concept word]
14.	care home.mp. [mp=title, book title, abstract, original title, name of substance word, subject heading word, floating sub-heading word, keyword heading word, organism supplementary concept word, protocol supplementary concept word, rare disease supplementary concept word, unique identifier, synonyms, population supplementary concept word, anatomy supplementary concept word]
15.	nursing home.mp. [mp=title, book title, abstract, original title, name of substance word, subject heading word, floating sub-heading word, keyword heading word, organism supplementary concept word, protocol supplementary concept word, rare disease supplementary concept word, unique identifier, synonyms, population supplementary concept word, anatomy supplementary concept word]
16.	advanced cancer.mp. [mp=title, book title, abstract, original title, name of substance word, subject heading word, floating sub-heading word, keyword heading word, organism supplementary concept word, protocol supplementary concept word, rare disease supplementary concept word, unique identifier, synonyms, population supplementary concept word, anatomy supplementary concept word]
17.	advanced dementia.mp. [mp=title, book title, abstract, original title, name of substance word, subject heading word, floating sub-heading word, keyword heading word, organism supplementary concept word, protocol supplementary concept word, rare disease supplementary concept word, unique identifier, synonyms, population supplementary concept word, anatomy supplementary concept word]
18.	end of life.mp. [mp=title, book title, abstract, original title, name of substance word, subject heading word, floating sub-heading word, keyword heading word, organism supplementary concept word, protocol supplementary concept word, rare disease supplementary concept word, unique identifier, synonyms, population supplementary concept word, anatomy supplementary concept word]
19.	hospice.mp. [mp=title, book title, abstract, original title, name of substance word, subject heading word, floating sub-heading word, keyword heading word, organism supplementary concept word, protocol supplementary concept word, rare disease supplementary concept word, unique identifier, synonyms, population supplementary concept word, anatomy supplementary concept word]
20.	life threatening illness.mp. [mp=title, book title, abstract, original title, name of substance word, subject heading word, floating sub-heading word, keyword heading word, organism supplementary concept word, protocol supplementary concept word, rare disease supplementary concept word, unique identifier, synonyms, population supplementary concept word, anatomy supplementary concept word]
21.	advanced metastasis.mp. [mp=title, book title, abstract, original title, name of substance word, subject heading word, floating sub-heading word, keyword heading word, organism supplementary concept word, protocol supplementary concept word, rare disease supplementary concept word, unique identifier, synonyms, population supplementary concept word, anatomy supplementary concept word]
22.	place of death.mp. [mp=title, book title, abstract, original title, name of substance word, subject heading word, floating sub-heading word, keyword heading word, organism supplementary concept word, protocol supplementary concept word, rare disease supplementary concept word, unique identifier, synonyms, population supplementary concept word, anatomy supplementary concept word]
23.	bereavement.mp. [mp=title, book title, abstract, original title, name of substance word, subject heading word, floating sub-heading word, keyword heading word, organism supplementary concept word, protocol supplementary concept word, rare disease supplementary concept word, unique identifier, synonyms, population supplementary concept word, anatomy supplementary concept word]
24.	Frailty.mp. [mp=title, book title, abstract, original title, name of substance word, subject heading word, floating sub-heading word, keyword heading word, organism supplementary concept word, protocol supplementary concept word, rare disease supplementary concept word, unique identifier, synonyms, population supplementary concept word, anatomy supplementary concept word]
25.	('end-stage' and ('cancer' or 'lung' or 'heart')).mp. [mp=title, book title, abstract, original title, name of substance word, subject heading word, floating sub-heading word, keyword heading word, organism supplementary concept word, protocol supplementary concept word, rare disease supplementary concept word, unique identifier, synonyms, population supplementary concept word, anatomy supplementary concept word]
26.	Palliative care.mp. or exp Palliative Care/

27.	bereavement.mp. or exp Bereavement/
28.	Hospices.mp. or exp Hospices/
29.	palliative medicine.mp. or exp Palliative Medicine/
30.	"Acceptance and Commitment Therapy".mp. or exp "Acceptance and Commitment Therapy"/
31.	exp "Acceptance and Commitment Therapy"/mt [Methods]
32.	Neoplasms/px [Psychology]
33.	cancer.mp. [mp=title, book title, abstract, original title, name of substance word, subject heading word, floating sub-heading word, keyword heading word, organism supplementary concept word, protocol supplementary concept word, rare disease supplementary concept word, unique identifier, synonyms, population supplementary concept word, anatomy supplementary concept word]
34.	("COPD" or "heart failure" or "renal failure" or "multiple sclerosis" or "MS" or "MND").mp. [mp=title, book title, abstract, original title, name of substance word, subject heading word, floating sub-heading word, keyword heading word, organism supplementary concept word, protocol supplementary concept word, rare disease supplementary concept word, unique identifier, synonyms, population supplementary concept word, anatomy supplementary concept word]
35.	dementia.mp. [mp=title, book title, abstract, original title, name of substance word, subject heading word, floating sub-heading word, keyword heading word, organism supplementary concept word, protocol supplementary concept word, rare disease supplementary concept word, unique identifier, synonyms, population supplementary concept word, anatomy supplementary concept word]
36.	8 or 9 or 10 or 12 or 13 or 14 or 15 or 16 or 17 or 18 or 19 or 20 or 21 or 22 or 23 or 24 or 25 or 32 or 33 or 34 or 35
37.	26 or 27 or 28 or 29 or 30 or 31
38.	7 and 36 and 37

Table 2: Types of healthcare professionals delivering ACT interventions

Level *	Description	Frequency	%
**Level 1**	All health and social care professionals (no training required)	0	0
**Level 2**	General Practitioners, social workers, nurse specialist	3	20
**Level 3**	Counsellors and psychological therapists	0	0
**Level 4**	Mental health specialists: psychiatrists and clinical psychologists	7	47
**Student**	Delivered by students	4	31
**Not Specified**		1	7
**Total**	All professionals	13	100

*classified according to the United Kingdom National Institute for Clinical Excellence (NICE) Four Tier Model for delivering psychological interventions [1].

Table 3: Results for most common outcome measures reported in ACT intervention papers

Outcome Measure	Number of Papers reporting Outcome Measure	Combined sample size	Sample Sizes	Number with significant improvements	Number with improvements were not significant	Number with no change	Number with worsening outcomes
Psychological Flexibility ***2-13***	12	617	n= 11, n= 55, n= 10, n=2, n=14, n= 50, n=313, n= 24, n= 31, n= 42, n=25, n=40	2	9	1	-
Anxiety ***6-9, 14, 15***	6	486	n=35, n=55, n=105, n=313, n=24 n=28, n=31	2	4	-	-
Depression ***6-9, 14, 15***	6	486	n=35, n=55, n=313, n=24, n=28, n=31	2	4	-	-
Distress ***2, 4, 6, 8, 10, 11, 15***	7	196	n=31, n=42, n=50, n=11, n=10, n=24, n=28	3	3	1	-
Valued Living ***3, 5-7, 9, 10, 12***	7	450	n=55, n=313, n=42, n=2, n=24, n=14, n=40	1	5	1	-
Mindfulness ***4, 9***	2	324	n=11, n=313	-	1	1	-
Grief & Anticipatory Grief ***3, 7***	2	57	n=2, n=55	-	2	-	-
Quality of Life ***2, 8, 12, 13***	4	106	n=10, n=31, n=40, n=25	1	2	1	-

Post-Traumatic Stress Symptoms [9]	1	313	n=313	1	-	-	-
Fear of Dying [14]	1	35	n=35	-	1	-	-
Sense of Life [14]	1	35	n=35	-	1	-	-
Emotional Control [8]	1	31	n=31	1	-	-	-
Worry [15]	1	28	n=28	1	-	-	-
Stress [13]	1	25	n=25	-	-	1	-
Wellbeing [13]	1	25	n=25	-	1	-	-
Thought Suppression [8]	1	31	n=31	1	-	-	-
Experience of illness [9]	1	313	n=313	-	1	-	-
Intolerance of Uncertainty [15]	1	28	n=28	-	1	-	-
Disengagement [8]	1	31	n=31	-	1	-	-
Pain/ Symptom Interference [3, 6, 11, 14, 16]	5	172	n=50, n=47, n=24, n=35, n=2	-	2	3	-
Fatigue Interference [6, 11, 12, 15, 16]	5	189	n=50, n=47, n=24, n=28, n=40	1	3	1	-
Sleep Interference [11, 12, 15, 16]	4	145	n=28, n-50, n=47, n=40	2	1	1	-

Table 4: Data on enrolment and retention data for intervention studies

Population Studied	Enrolment Rate Range/ %	Enrolment Rate Highest/ Lowest		Retention Rate Range/ %	Retention Rate Highest/ Lowest	
**People with advanced progressive illness (n=7)** [2, 6, 8, 10, 14-17]	26-82	82%<br>Individual in-person intervention. Recruitment in clinics by the researcher [8].	26%<br>Individual telephone based intervention. Recruitment through mailed letters [6].	43-88	88%<br>Individual telephone based intervention consisting of 45 minute sessions delivered over 4 weeks. [6].	43%<br>Individual and in-person intervention consisting of 8 weekly 60 minute sessions [10].
**Informal caregivers (n=4)** [4, 5, 7, 9]	36-82	82%<br>Group in person intervention- 5x 90 minute sessions. Participants self-referred in response to promotional flyers and recommendations from clinicians [4].	36%<br>Group Videocall intervention- 6x 90 minute sessions. Recruited participants via telephone or on the hospital wards by the research team [9].	48-71	71%<br>Paper had lowest enrolment rate [9]	48%<br>Paper had highest enrolment rate [4]
**Bereaved and staff** [3]	No data provided	No data provided		No data provided	No data provided	
**Staff**[13]	No data provided	No data provided		60	15/25 completed the focus group follow up. Individual and group videocall intervention.	
**Mixed- people with advanced progressive illness and their caregivers** [11, 12]	51-54	51%<br>Telephone and mixed (group and individual) intervention consisting of six 60 minute sessions [11]	54%<br>Telephone and mixed (group and individual) intervention consisting of six 60 minute sessions [12]	73-76	73 [12]	76 [11]

Table 5: Data on satisfaction rate for intervention studies

Population	Satisfaction Data
**People with advanced progressive illness [2, 6, 8, 10, 14-16]**	• Only four of the papers reported on the satisfaction rates of their interventions (n=4) [2, 6, 10, 14].<br>• Interventions were generally acceptable [2, 6, 10, 14].<br>• Participants commented that they liked the environment of the hospice for intervention delivery but found the practical components of the intervention difficult and the language used too complex [2].<br>• An 8-session intervention was too much of a commitment [10].
**Informal caregivers [4, 5, 7, 9]**	• Intervention was generally acceptable, commenting that the tailored telephone component was more useful in contrast to the generalised leaflet [7].<br>• Participants liked the clear structure, however some found it did not meet their needs [5].
**Staff [13]**	• Participants gave positive feedback overall.<br>• Participants noted high quality of facilitation of group sessions.<br>• Participants found that the online aspect did not impede the ability to feel like they were in a safe place to open up about their experiences.<br>• They found the workbook helped to keep them on track each week and that the self-directed modules gave flexibility
**Bereaved [3]**	No data provided
**Mixed [11, 12]**	No data provided

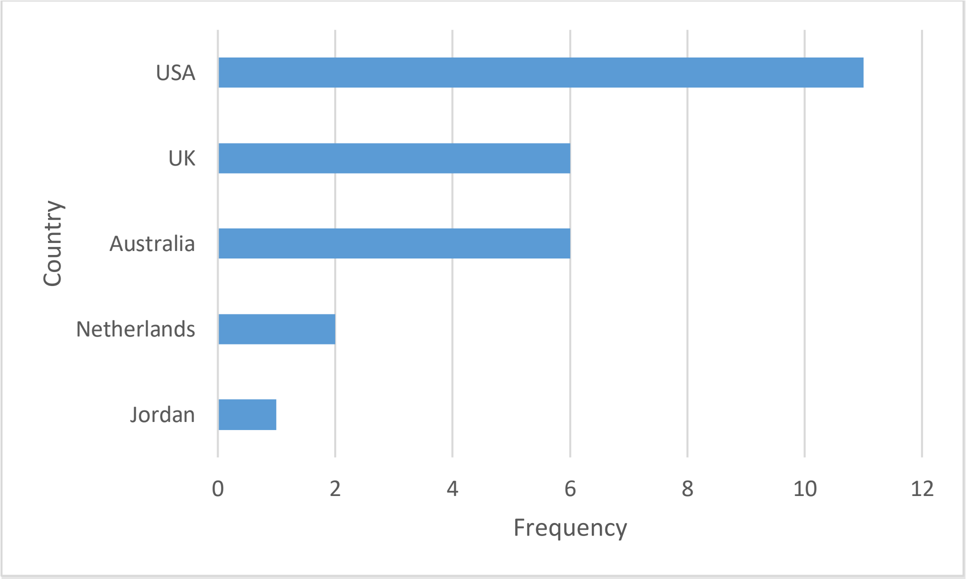

<u>Figure 1:</u> Scatter graph illustrating country of publication for included studies (n=26)

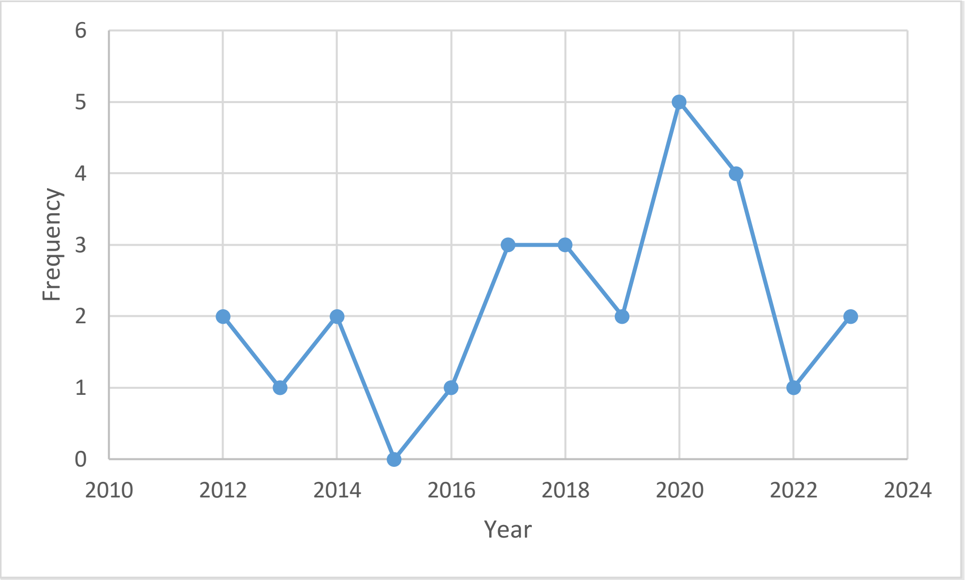

<u>Figure 2:</u> Scatter graph illustrating years of publication for the included studies (n=26)

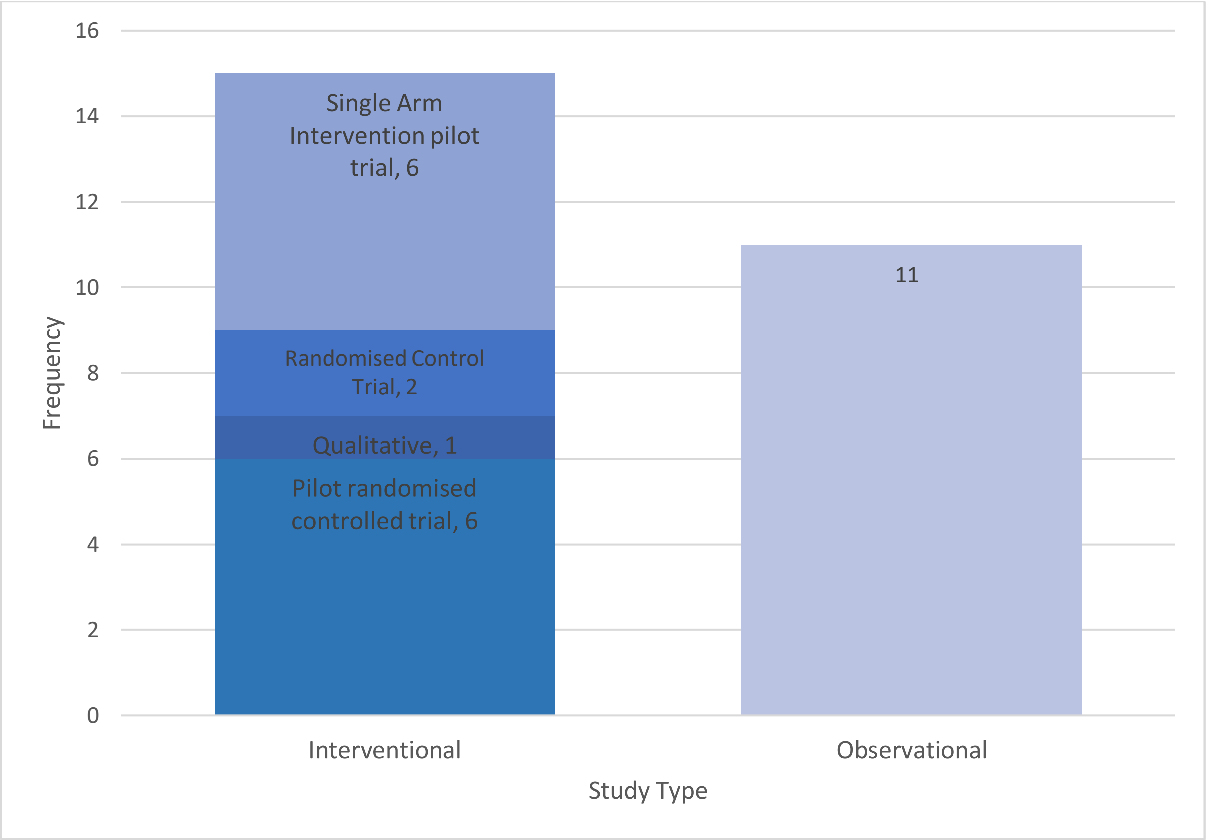

Figure 3: Stacked bar chart for study type of included studies (n=26)

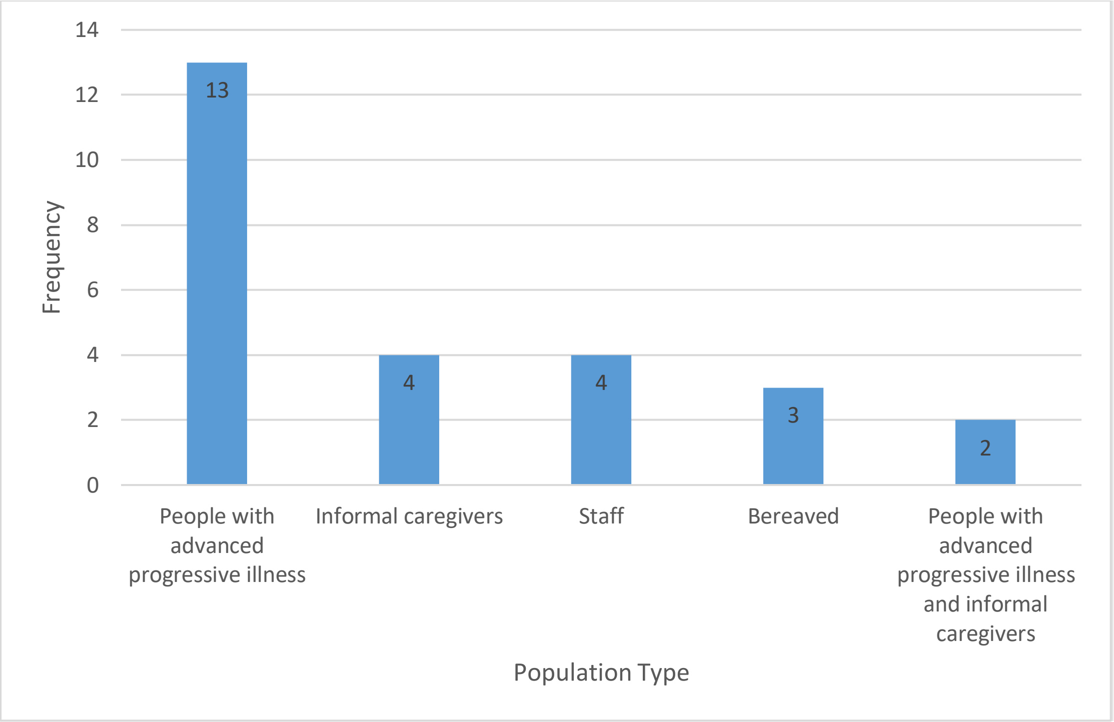

<u>Figure 4:</u> Study populations for included studies.

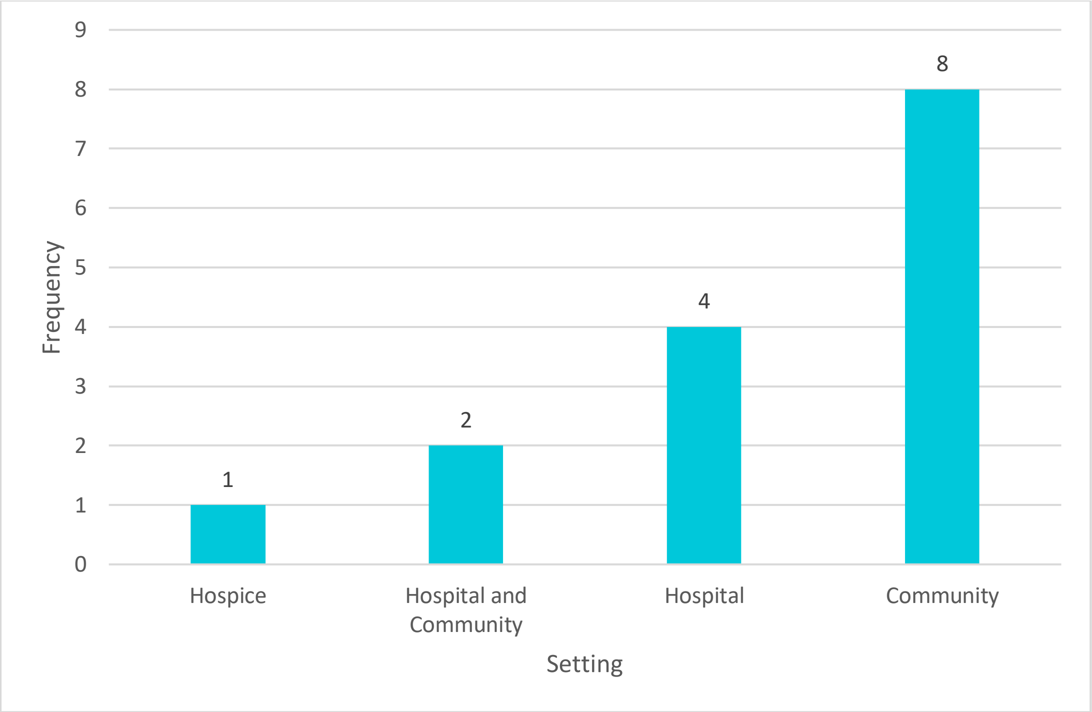

Figure 5: Study settings for intervention studies (n=15)

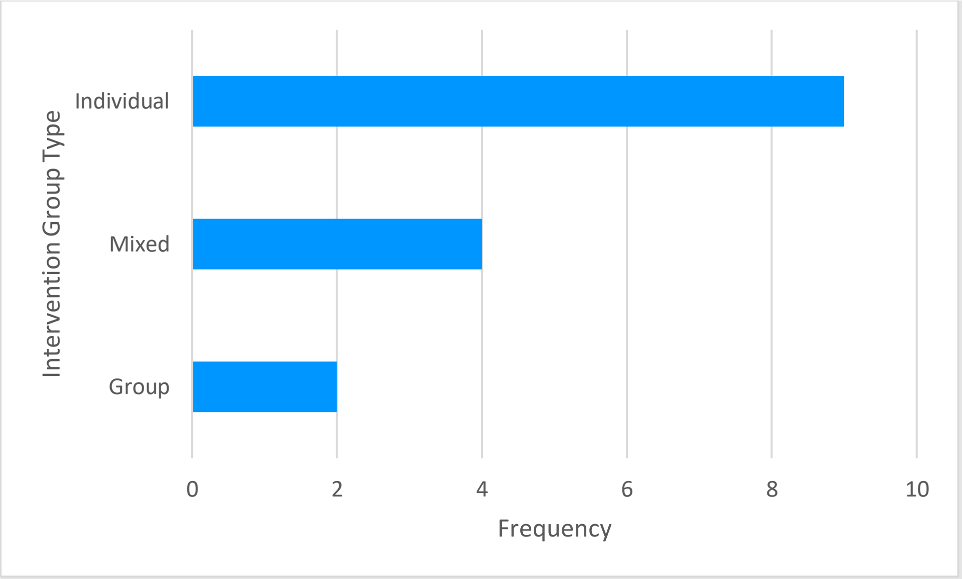

Figure 6: Intervention format for intervention studies (n=15)

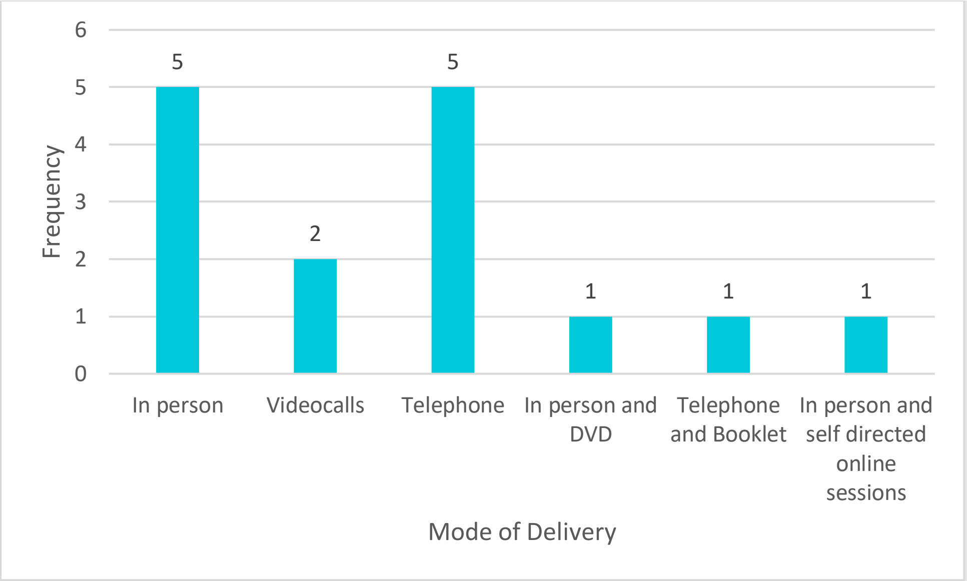

Figure 7: Mode of delivery of Acceptance and Commitment Therapy interventions (n=15)

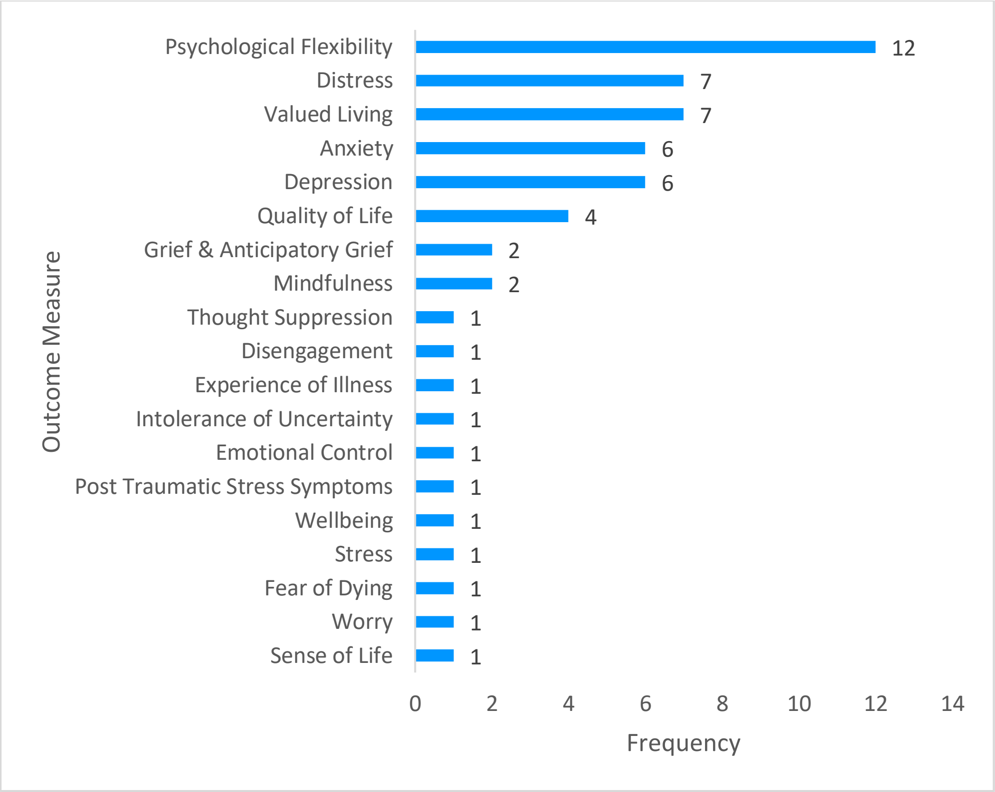

Figure 8: Frequency of psychological outcome measures used in intervention studies (N=15)

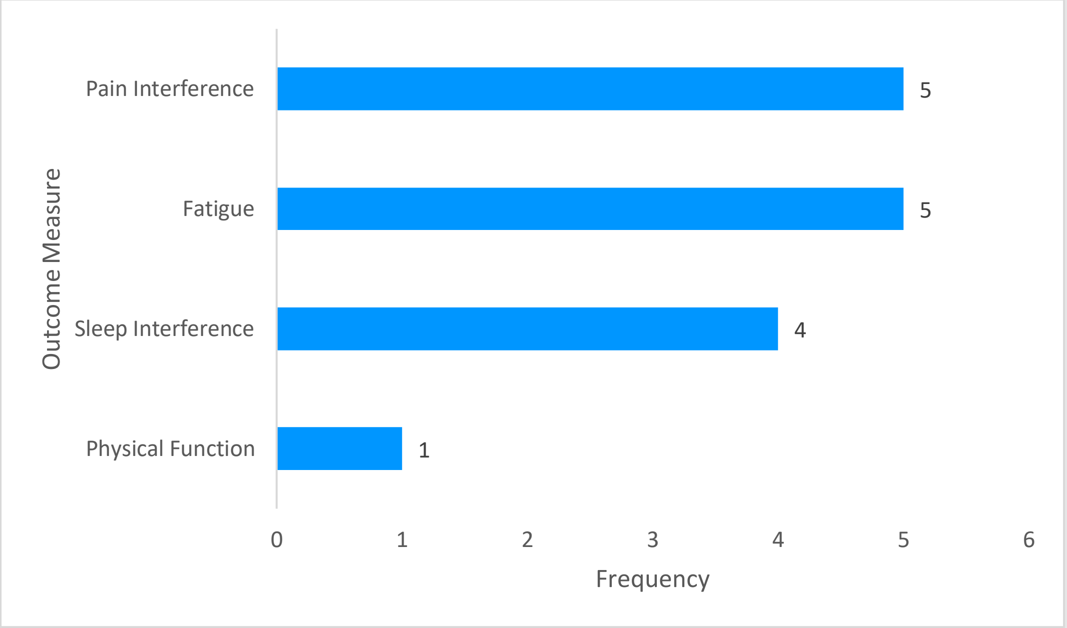

Figure 9: Frequency of physical outcome measures

Supplementary Material References:

1. National Institute for Health and Clinical Excellence. Guidance on cancer services: Improving supportive and palliative care for adults with adults the manual. In: Excellence NIfC, (ed.). London2004.
2. Hulbert-Williams NJ, Norwood SF, Gillanders D, et al. Brief Engagement and Acceptance Coaching for Hospice Settings (the BEACHeS study): results from a Phase I study of acceptability and initial effectiveness in people with non-curative cancer. *BMC Palliative Care* 2021; 20: 1-13. DOI: 10.1186/s12904-021-00801-7.
3. Johnson JL. A Pilot study of Acceptance and Commitment Therapy (ACT) for Prolonged Grief Disorder (PGD). Wright Institute Graduate School of Psychology, ProQuest, 2014.
4. Burke K, Muscara F, McCarthy M, et al. Adapting acceptance and commitment therapy for parents of children with life-threatening illness: Pilot study. *Families, Systems, & Health* 2014; 32: 122-127.
5. Kohle N, Drossaert CHC, Jaran J, et al. User-experiences with a web-based self-help intervention for partners of cancer patients based on acceptance and commitment therapy and self-compassion: a qualitative study. *BMC Public Health* 2017; 17: 225.
6. Plumb Vilardaga JC, Winger JG, Teo I, et al. Coping Skills Training and Acceptance and Commitment Therapy for Symptom Management: Feasibility and Acceptability of a Brief Telephone-Delivered Protocol for Patients With Advanced Cancer. *Journal of Pain and Symptom Management* 2020; 59: 270-278. DOI: 10.1016/j.jpainsymman.2019.09.005.
7. Davis EL, Deane FP, Lyons GC, et al. Feasibility randomised controlled trial of a self-help acceptance and commitment therapy intervention for grief and psychological distress in carers of palliative care patients. *Journal of Health Psychology* 2020; 25: 322-339. DOI: 10.1177/1359105317715091.
8. Rost AD, Wilson K, Buchanan E, et al. Improving Psychological Adjustment Among Late-Stage Ovarian Cancer Patients: Examining the Role of Avoidance in Treatment. *Cognitive and Behavioral Practice* 2012; 19: 508-517. DOI: 10.1016/j.cbpra.2012.01.003.
9. Muscara F, McCarthy MC, Rayner M, et al. Effect of a Videoconference-Based Online Group Intervention for Traumatic Stress in Parents of Children With Life-threatening Illness: A Randomized Clinical Trial. *JAMA Network Open* 2020; 3: e208507. Randomized Controlled Trial
Research Support, Non-U.S. Gov't.
10. Serfaty M, Armstrong M, Vickerstaff V, et al. Acceptance and commitment therapy for adults with advanced cancer (CanACT): A feasibility randomised controlled trial. *Psycho-Oncology* 2019; 28: 488-496.
11. Mosher CE, Secinti E, Hirsh AT, et al. Acceptance and Commitment Therapy for symptom interference in advanced lung cancer and caregiver distress: A pilot randomized trial. *Journal of Pain and Symptom Management* 2019; 58: 632-644.
12. Mosher CE, Secinti E, Wu W, et al. Acceptance and commitment therapy for patient fatigue interference and caregiver burden in advanced gastrointestinal cancer: Results of a pilot randomized trial. *Palliative medicine* 2022; 36: 1104-1117. DOI: 10.1177/02692163221099610.
13. Finucane AM, Hulbert-Williams NJ, Swash B, et al. Feasibility of RESTORE: An online Acceptance and Commitment Therapy intervention to improve palliative care staff wellbeing. *Palliative Medicine* 2023; 37: 244-256. DOI: 10.1177/02692163221143817.
14. Arch JJ, Fishbein JN, Ferris MC, et al. Acceptability, Feasibility, and Efficacy Potential of a Multimodal Acceptance and Commitment Therapy Intervention to Address Psychosocial and Advance Care Planning Needs among Anxious and Depressed Adults with

Metastatic Cancer. *Journal of Palliative Medicine* 2020; 23: 1380-1385. DOI: 10.1089/jpm.2019.0398.
15. Wells-Di Gregorio SM, Marks DR, DeCola J, et al. Pilot randomized controlled trial of a symptom cluster intervention in advanced cancer. *Psycho-Oncology* 2018; 28: 76-84.
16. Mosher CE, Secinti E, Li R, et al. Acceptance and commitment therapy for symptom interference in metastatic breast cancer patients: a pilot randomized trial. *Supportive Care in Cancer* 2018; 26: 1993-2004. Randomized Controlled Trial.
17. Mosher CE, Tometich DB, Hirsh A, et al. Symptom experiences in metastatic breast cancer patients: Relationships to activity engagement, value-based living, and psychological inflexibility. *Psycho-Oncology* 2017; 26: 1944-1951.
